# Mitochondrial hormesis links low-dose arsenite exposure to lifespan extension

**DOI:** 10.1111/acel.12076

**Published:** 2013-05-06

**Authors:** Sebastian Schmeisser, Kathrin Schmeisser, Sandra Weimer, Marco Groth, Steffen Priebe, Eugen Fazius, Doreen Kuhlow, Denis Pick, Jürgen W Einax, Reinhard Guthke, Matthias Platzer, Kim Zarse, Michael Ristow

**Affiliations:** 1 Department of Human Nutrition, Institute of Nutrition, University of JenaD-07743, Jena, Germany; 2 Leibniz Graduate School of Aging, Leibniz Institute for Age Research, Fritz-Lipmann-InstituteD-07745, Jena, Germany; 3 Department of Clinical Nutrition, German Institute of Human Nutrition Potsdam-RehbrückeD-14558, Nuthetal, Germany; 4 Energy Metabolism Laboratory, Swiss Federal Institute of Technology (ETH) ZurichSchwerzenbach/Zürich, CH 8603, Switzerland; 5 Genome Analysis Group, Leibniz Institute for Age Research, Fritz-Lipmann-InstituteD-07745, Jena, Germany; 6 Systems Biology and Bioinformatics Group, Leibniz Institute for Natural Product Research and Infection Biology, Hans-Knöll-InstituteD-07745, Jena, Germany; 7 Department of Environmental Analysis, Institute of Inorganic and Analytical Chemistry, University of JenaD-07743, Jena, Germany

**Keywords:** arsenite, *Caenorhabditis elegans*, hormesis, lifespan, mitochondria, mitohormesis, oxidative stress, reactive oxygen species, ROS, toxicology

## Abstract

Arsenite is one of the most toxic chemical substances known and is assumed to exert detrimental effects on viability even at lowest concentrations. By contrast and unlike higher concentrations, we here find that exposure to low-dose arsenite promotes growth of cultured mammalian cells. In the nematode *C. elegans*, low-dose arsenite promotes resistance against thermal and chemical stressors and extends lifespan of this metazoan, whereas higher concentrations reduce longevity. While arsenite causes a transient increase in reactive oxygen species (ROS) levels in *C. elegans*, co-exposure to ROS scavengers prevents the lifespan-extending capabilities of arsenite, indicating that transiently increased ROS levels act as transducers of arsenite effects on lifespan, a process known as mitohormesis. This requires two transcription factors, namely DAF-16 and SKN-1, which employ the metallothionein MTL-2 as well as the mitochondrial transporter TIN-9.1 to extend lifespan. Taken together, low-dose arsenite extends lifespan, providing evidence for nonlinear dose-response characteristics of toxin-mediated stress resistance and longevity in a multicellular organism.

## Introduction

Arsenic is a ubiquitous metalloid derived from natural and anthropogenic sources that occurs in inorganic as well as organic forms. The major inorganic forms of arsenic (As) include trivalent meta-arsenite (As^3+^, ‘arsenite’) and pentavalent arsenate (As^5+^) (World-Health-Organisation, 2004). Trivalent arsenic is known to be more toxic than the pentavalent form (World-Health-Organisation, 2004).

There is a general scientific consensus that arsenite is acting as a toxin even at lowest concentrations (World-Health-Organisation, 2004), implicating linear dose-response characteristics. In particular, arsenite has been linked to promotion of a number of metabolic diseases, including not only diabetes, cardiovascular and neurodegenerative disorders, but also various cancers (World-Health-Organisation, 2004). The most significant source of exposure is contaminated drinking water (World-Health-Organisation, 2004). The underlying mechanisms for arsenite-induced diseases have been extensively studied but nevertheless appear to lack a common denominator. Exposure to arsenite has been repeatedly affiliated to impaired DNA repair, alterations in DNA methylation status, impaired mitochondrial integrity, and increased levels of oxidative stress (Flora, 2012).

Despite their toxic nature, arsenite-containing agents have been in medicinal use for several millennia (Waxman & Anderson, 2001) and are currently being used in traditional Chinese as well as homeopathic medicine (Kerr & Saryan, 1986; Thomas & Troncy, 2009) the latter, however, at typically undetectable concentrations.

One of the better-known examples, Fowler's solution, contains 1% potassium arsenite and was in medicinal use until the 1950s for multiple diseases, as a general tonic and to change skin complexity (Waxman & Anderson, 2001). Recently, arsenite has been approved as a chemotherapeutic agent for the treatment of acute promyelocytic leukemia (Nicolis *et al*., 2009).

Most interestingly, recent epidemiological evidence indicates that exposure to low doses of environmental arsenite may unexpectedly be associated with decreased cancer risk (Karagas *et al*., 2002; Lamm *et al*., 2004; Schoen *et al*., 2004; Baastrup *et al*., 2008). These findings implicitly question linear dose-response characteristics between arsenite exposure and disease risk.

Taken together, the above-mentioned findings tentatively suggest that arsenite may exert health-promoting effects at low doses due to unknown mechanisms, while the same compound acts as a lifespan-shortening toxin at higher doses. We here systematically study the effects of low-dose arsenite on metabolic health and longevity in two different model systems, that is, cultured cells and the nematode *C. elegans*.

## Results

### Low-dose arsenite promotes growth of cultured cells

**Fig. 1:**
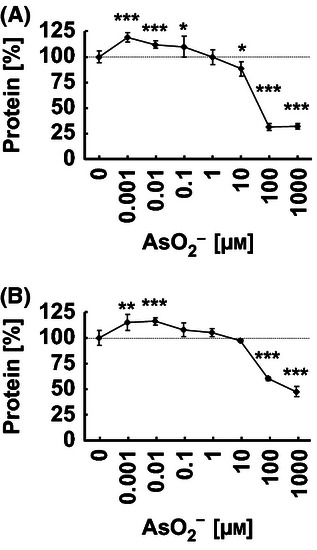
Low-dose arsenite promotes growth of cultured cells. (A) Effects of arsenite exposure for 24 h on growth of murine NIH 3T3 fibroblasts. (B) Results of identical experimental setup using human HepG2 hepatoma cells.

To study the putative effects of low-dose arsenite on mammalian model systems, we first have exposed two cell lines, murine NIH-3T3 fibroblasts and human HepG2 hepatoma cells, to increasing concentrations of arsenite. While higher concentrations of arsenite predictably impair growth of both cell lines (Fig. 1), lower concentrations of 1–10 nm promote growth of these cells (Fig. 1). These findings indicate that there is a nonlinear dose-response pattern to arsenite exposure, where low concentrations exert the opposite effect of higher concentrations. This phenomenon has been named hormesis and was proposed more than a century ago to act as the functional basis of most pharmaceutically used drugs (Calabrese & Baldwin, 2003; Kaiser, 2003).

### Low-dose arsenite promotes stress resistance of nematodes and extends *C. elegans* lifespan

**Fig. 2:**
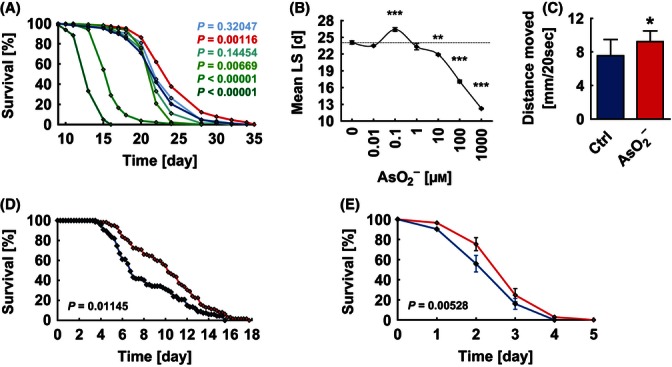
Low-dose arsenite promotes lifespan and stress resistance in the roundworm *C. elegans*. (A) Typical effects of different concentrations of arsenite on *C. elegans* lifespan (crimson blue: control, light blue: 10 nm, red: 100 nm, blue-green: 1 μm, light green: 10 μm, crimson green: 100 μm, dark green: 1 mm of arsenite, respectively). (B) Effects of different concentrations of arsenite on *C. elegans* mean lifespan (see Table 1 for number of independent experiments). (C) Locomotor activity of nematodes in the absence (blue) and presence of 100 nm arsenite (red). (D) Survival of untreated (blue) and arsenite pretreated (red) nematodes with subsequent exposure to heat stress. (E) Survival of untreated (blue) and arsenite pretreated (red) nematodes with subsequent exposure to paraquat stress.

We next tested whether arsenite may affect lifespan of the short-lived multicellular model organism *C. elegans*. Very similar to our findings in cultured cells (Fig. 1), we observed a biphasic dose-response of nematodes to arsenite. In particular, life-long exposure of adult nematodes to arsenite concentrations of 10 μm and above predictably decreased median lifespan (Fig. 2A,B) (Table 1, also applies to all subsequent lifespan assays), while a concentration of 100 nm surprisingly extends median lifespan by 10 percent (Fig. 2A,B) (Fig. S1 for individual results). The corresponding concentrations of arsenic in whole worm-lysates are given in Fig. S2A (Supporting information).

**Table 1:** Lifespan assay results and statistical analyses

Strain, treatment	Maximum lifespan (Days ± SEM)	Mean lifespan (Days ± SEM)	*P*-value vs. control*	Number of experiments	Number of nematodes
N2 control	33.9 ± 0.6	24.1 ± 0.3	n.a.	6	637
N2 + 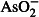 0.01 μm	34.0	23.5 ± 0.1	0.4261	3	352
N2 + 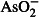 0.1 μm	37.8 ± 0.6	26.4 ± 0.3	0.0048	6	665
N2 + 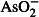 1 μm	32.0	23.3 ± 0.5	0.3554	3	354
N2 + 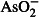 10 μm	28.0	21.9 ± 0.1	0.0057	3	353
N2 + 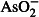 100 μm	23.0	16.8 ± 0.3	< 0.0001	3	370
N2 + 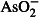 1000 μm	17.0	12.4 ± 0.1	< 0.0001	3	349
N2 + BHA	35.0	24.6 ± 0.2	0.2525 (n.s.)	3	354
N2 + BHA + 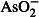 0.1 μm	35.0 ± 0.6	24.0 ± 0.2	0.8382 (n.s.)	3	375
N2 + NAC	34.5 ± 0.5	23.9 ± 0.2	0.8489 (n.s.)	2	161
N2 + NAC + 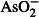 0.1 μm	34.5 ± 0.5	24.3 ± 0.4	0.6664 (n.s.)	2	170
*skn-1*	27.7 ± 1.3	17.8 ± 0.1	n.a.	3	256
*skn-1* + 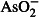 0.1 μm	24.3 ± 0.6	17.0 ± 0.2	0.0245	3	262
*daf-16*	33.0	22.9 ± 0.1	n.a.	3	409
*daf-16* + 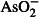 0.1 μm	31.7 ± 1.2	22.1 ± 0.1	0.0125	3	397
*mtl-2*	34.8 ± 0.5	24.1 ± 0.1	n.a.	4	432
*mtl-2* + 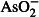 0.1 μm	37.8 ± 1.0	24.5 ± 0.3	0.0722	4	429
*tin-9.1*	35.3 ± 1.0	23.0 ± 0.2	n.a.	4	593
*tin-9.1* + 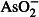 0.1 μm	35.8 ± 0.3	23.0 ± 0.2	0.7958	4	629

* *t*-test of mean lifespan.

n.a., not applicable; n.s., not significant; BHA, butylated hydroxyanisole

In addition to its lifespan-extending effects (Fig. 2A,B) when exposing adult nematodes to 100 nm of arsenite, we observe increased locomotor activity (Fig. 2C) and increased resistance against thermal stress (Fig. 2D) as well as against the catalytic free radical generator paraquat (Fig. 2E) (Fig. S1 for individual results), indicating increased resistance against oxidative stress. However, arsenite had no effect on food uptake (Fig. S2B) and, unlike the positive-control paraquat, did not cause an aversion response (Fig. S2C,D).

Taken together, these findings indicate that arsenite exerts a biphasic effect on mean lifespan, and in particular that low-dose arsenite unexpectedly extends lifespan and stress resistance in a metazoan.

### Low-dose arsenite extends lifespan by transiently increasing oxidative stress

**Fig. 3:**
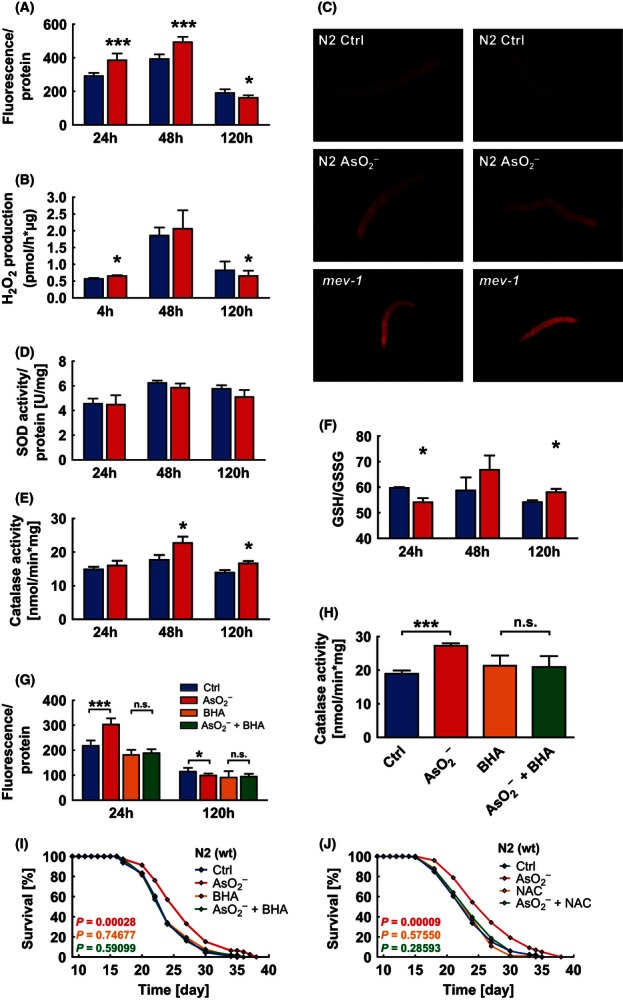
Low-dose arsenite promotes ROS formation and subsequently ROS defense in *C. elegans*. (A) Mitochondrial ROS formation following exposure to arsenite for different time periods (blue: control wild-type worms, red: wild-type worms exposed to 100 nm arsenite). (B) Formation of hydrogen peroxide in nematode media following exposure to arsenite for different time periods (blue: control wild-type worms, red: wild-type worms exposed to 100 nm arsenite). (C) Fluorescent microphotographs of MitoTracker Red CM-H_2_X-treated nematodes, (D) superoxide dismutase activities, (E) catalase activities, and (F) ratios of reduced glutathione to oxidized glutathione, all in control worms (blue) and nematodes exposed to 100 nm arsenite (red). (G) Mitochondrial ROS formation following exposure to arsenite and butylated hydroxyanisole (BHA) for different time periods (blue: control, red: worms exposed to 100 nm arsenite, orange: worms exposed to 10 μm BHA, green: worms exposed to 100 nm arsenite and 10 μm BHA). (H) Catalase activities measured after 120 hrs incubation period (blue: control, red: worms exposed to 100 nm arsenite, orange: worms exposed to 10 μm BHA, green: worms exposed to 100 nm arsenite and 10 μm BHA). (I) Lifespan data for wild-type worms in the absence of arsenite and in the absence of BHA (blue), in the presence of 100 nm arsenite and in the absence of BHA (red), in the absence of arsenite but in the presence of 10 μm BHA (orange), and lastly the presence of both agents (green). (J) Lifespan data for wild-type worms in the absence of arsenite and in the absence of *N*-acetyl-*L*-cysteine (NAC) (blue), in the presence of 100 nm arsenite and in the absence of NAC (red), in the absence of arsenite but in the presence of 1 mm NAC (orange), and lastly the presence of both agents (green).

While multiple molecular effects of arsenite toxicity have been accumulated in the literature, increased oxidative stress by inadequate detoxification of reactive oxygen species (ROS) or increased formation of these has been repeatedly observed (Smith *et al*., 2001; Miller *et al*., 2002; Shi *et al*., 2004; Cui *et al*., 2008). Hence, we have analyzed ROS levels in adult nematodes that were exposed to 100 nm arsenite, in a time-resolved manner using two independent and mechanistically distinct methods. Both methods indicate that arsenite exposure causes an early and transient increase in nematodal ROS (Fig. 3A,B), whereas ROS levels were significantly decreased in the steady state, that is, after exposure to arsenite for 5 days (Fig. 3A,B). The constitute *mev-1* mutant known to exert increased ROS levels (Senoo-Matsuda *et al*., 2001) served as a positive control (Fig. S3A,B). It should be noted that *mev-1* nematodes are short-lived (Senoo-Matsuda *et al*., 2001), linked to the fact that the increase in ROS levels in *mev-1* relative to N2 is at least 5-fold higher than the ROS increase following arsenite exposure (Fig. S3A,B). Moreover, *mev-1* ROS levels are permanently increased, whereas arsenite-induced ROS is detectable for a limited time period only (Fig. 3A,B). Representative nematodes derived from Fig. 3A and Fig. S3A (Supporting information), respectively, are depicted in Fig. 3C. Next, we quantified activities of the two key enzymes of ROS detoxification in *C. elegans*, superoxide dismutase (Fig. 3D), and catalase (Fig. 3E). The latter was found to be induced following the primary increase in ROS (Fig. 3A,B) suggesting that increased catalase activity is a response to primarily increased ROS levels, causing a reduction of ROS load in the steady state. A similar pattern was observed for the ratio of reduced to oxidized glutathione (Fig. 3F), altogether suggesting that the initial increase in ROS following arsenite exposure may be essential for arsenite effects on lifespan. To test this, we have pre-incubated nematodes with the antioxidant butylated hydroxyanisole (BHA), which efficiently blocks accumulation of ROS in nematodes (Zarse *et al*., 2012). Consistently, arsenite was unable to induce ROS in the presence of BHA (Fig. 3G). Importantly, the induction of catalase activity by arsenite-mediated ROS (Fig. 3E) was similarly abolished in the presence of BHA (Fig. 3H), indicating that the initial ROS signal is required for the secondary induction of catalase activity.

When exposing BHA-treated worms to arsenite at a concentration of 100 nm, no extension of lifespan was observed (Fig. 3I). Similar results were obtained for a mechanistically independent antioxidant, *N*-acetyl-*L*-cysteine (NAC), that also abolished the effects of low-dose arsenite on lifespan (Fig. 3J) both indicating that ROS formation induced by arsenite in the absence of antioxidants only is responsible for lifespan extension in *C. elegans*. A similar mechanism, sometimes named mitochondrial hormesis or mitohormesis, has been found in *C. elegans* (Schulz *et al*., 2007; Doonan *et al*., 2008; Lee *et al*., 2010; Yang & Hekimi, 2010; Zarse *et al*., 2012), *S. cerevisae* (Mesquita *et al*., 2010; Pan *et al*., 2011), *D. melanogaster* (Albrecht *et al*., 2011), and humans (Ristow *et al*., 2009).

### Low-dose arsenite promotes transcriptional activity of SKN-1/NRF-2 and DAF-16/FOXO

**Fig. 4:**
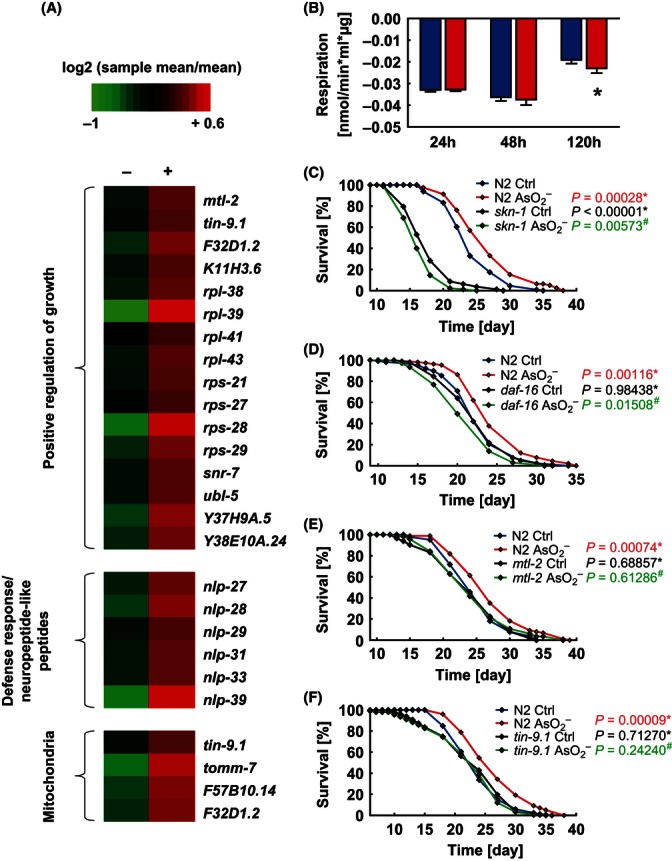
RNA deep sequencing reveals transcriptional mediators and targets of low-dose arsenite exposure. (A) Functional groups of differentially expressed messenger RNAs following exposure with 100 nm arsenite for 48 hrs in *C. elegans*. (B) Time-resolved quantification of oxygen consumption in control worms (blue) and nematodes exposed to 100 nm arsenite (red). (C–F) Lifespan analyses using mutant nematodes for *skn-1(zu67)* (C), *daf-16(mgDf47)* (D), *mtl-2(gk125)* (E), and lastly *tin-9.1(ok2194)* (F) in the absence of arsenite (black) and in the presence of 100 nm arsenite (green) in parallel to wild-type *C. elegans* (blue: control, red: 100 nm arsenite; * *P*-value refers to N2 control; # *P*-value refers to the corresponding mutant control).

Next, we have quantified all genes differentially expressed following exposure to arsenite for 48 hrs in comparison with untreated worms by RNA deep sequencing. Analyzing these genes by clustering them into functional groups (Fig. 4A) (Tables S2 and S3) revealed that, besides others, growth-promoting and mitochondrial proteins are upregulated following exposure to arsenite (Fig. 4A). Consistent with the fact that arsenite exposure causes an induction of mitochondrial proteins (Fig. 4A), we observed an induction of mitochondrial activity, namely respiration, following exposure to arsenite (Fig. 4B). We next analyzed all upregulated genes (Table S1) regarding the prevalence of transcription factors consensus motifs in the promoter regions. We found the motifs for SKN-1 (Fig. S4A,B), an orthologue of the KEAP-1-associated, mammalian transcription factor NRF-2, or DAF-16 (Fig. S4C), an orthologue of the mammalian FOXO transcription factor family, in 67.1 and 48.2% of arsenite-induced genes, respectively (Fig. S4D). This suggests that the vast majority of arsenite-induced genes may depend on activation of SKN-1 or DAF-16 (Oliveira *et al*., 2009), whereas no differences in the expression levels of both transcription factors was observed [mean counts SKN-1: 16145.27 (ctrl) vs. 16943.67 (arsenite); mean counts DAF-16: 23275.56 (ctrl) vs. 22761.23 (arsenite)].

However, when analyzing nematodes deficient for either SKN-1 or DAF-16, we observed, unlike in wild-type worms, a reduction in lifespan following exposure to arsenite at a concentration of 100 nm, indicating that both transcription factors independently are required for arsenite-mediated promotion of longevity (Fig. 4C,D). Lastly, we tested whether genes that have been identified by RNA sequencing (Fig. 4A) are essential for the longevity-promoting effects of arsenite in *C. elegans*. By exposing constitutive mutants for the metallothionein *mtl-2* as well as a mitochondrial transporter *tin-9.1*–100 nm of arsenite, that is, the concentration that has been established to extend lifespan in wild-type worms in the current study (Fig. 2A,B), no promotion of longevity could be observed in either mutant (Fig. 4E,F), indicating that both genes are required for the extension of lifespan by arsenite. Consistently, we identified a SKN-1 binding site in the promoter region of *tin-9.1*. For *mtl-2*, we found several SKN-1 and DAF-16 binding sites in the promoter region, suggesting that the expression of both genes is under control of SKN-1 and DAF-16.

These latter findings indicate that low-dose arsenite induces a transient ROS signal to promote transcriptional activity of both DAF-16 and SKN-1 culminating in induction of specific mitochondrial and stress response genes that are essential for the lifespan-extending capabilities of low-dose arsenite.

## Discussion

In the present study, we have shown that arsenite promotes cellular growth and exerts lifespan-extending properties by transiently increasing oxidative stress. There is ample published evidence that arsenite promotes formation of ROS by induction of mitochondrial (Miller *et al*., 2002; Shi *et al*., 2004) and nonmitochondrial (Smith *et al*., 2001; Cui *et al*., 2008) processes. This increased formation of ROS following arsenite exposure has been linked to a number of arsenite-associated diseases, including diabetes mellitus (Navas-Acien *et al*., 2008) and various cancers (Druwe & Vaillancourt, 2010), as well as neurodegenerative (Vahidnia *et al*., 2007) and cardiovascular (Navas-Acien *et al*., 2005) disorders.

Nevertheless, a number of recent reports suggest that exposure to low-dose arsenite may be associated with decreased incidences of various cancers in humans (Karagas *et al*., 2002; Lamm *et al*., 2004; Schoen *et al*., 2004; Baastrup *et al*., 2008). In this regard, it may be interesting to note that arsenite is a relevant constituent of medieval European as well as traditional Chinese medicine (Miller *et al*., 2002). Accordingly, previous reports suggest that low-dose arsenite may protect from DNA damage and may increase DNA repair (Snow *et al*., 2005), induce telomerase activity (Zhang *et al*., 2003) and NRF-2-dependent stress defence capacities (Pi *et al*., 2003), increase reduced cellular glutathione (Deneke, 1992), and lastly promote cellular proliferation (Yang *et al*., 2007). Notably, these findings are consistent with our current observation and may be considered necessary constituents of increased lifespan following arsenite exposure.

Consistent with an essential role for arsenite in inducing ROS formation (Smith *et al*., 2001; Cui *et al*., 2008), our findings indicate that increased oxidative stress is crucial for increased longevity following low-dose arsenite exposure. For other interventions, including calorie restriction, hypoxia, reduced mTOR signaling, impaired expression of antioxidant defense enzymes as well as components of the respiratory chain, and lastly physical exercise, a similar role for increased ROS formation has been observed, notably in different model systems, including *C. elegans* (Schulz *et al*., 2007; Doonan *et al*., 2008; Lee *et al*., 2010; Yang & Hekimi, 2010; Zarse *et al*., 2012), *S. cerevisae* (Mesquita *et al*., 2010; Pan *et al*., 2011), *D. melanogaster* (Albrecht *et al*., 2011), and humans (Ristow *et al*., 2009). The transient nature of the ROS signal that is required to extend lifespan has been recently observed in states of impaired insulin-/IGF1-signaling (Zarse *et al*., 2012). Taken together, these findings suggest that rather diverse interventions, listed within this paragraph, may culminate in both increased ROS formation as well as promotion of lifespan and metabolic health, respectively, as discussed in more detail elsewhere (Ristow & Schmeisser, 2011).

In summary, our findings provide evidence that low-dose exposure to the toxin arsenite extends metazoan lifespan through activation of mitochondrial ROS formation and subsequent defense mechanisms resembling a process named mitochondrial hormesis or mitohormesis (Ristow & Schmeisser, 2011).

## Experimental procedures

### Cell culture experiments/conditions

HepG2 and NIH-3T3 cells were obtained from ATCC (American Type Culture Collection). HepG2 cells were maintained in Roswell Park Memorial Institute (RPMI) medium (Pan-Biotech, Aidenbach, Germany) containing 10% (v/v) FBS (Biochrom AG, Berlin, Germany) and 1% (v/v) antibiotic solution (100 U mL^−1^ penicillin, and 10 μg mL^−1^ streptomycin (P/S), obtained from Biochrom AG, Berlin, Germany) like previously described. NIH-3T3 cells were maintained in Dulbecco's modified Eagle's medium (DMEM; Cambrex Bio Science, Verviers, Belgium) containing 10% (v/v) FBS and 1% (v/v) antibiotic solution. Both cell lines were cultivated in humidified atmosphere of 5% CO_2_ at 37 °C.

### Cell culture experiments/determination of cell growth

To determine growth in human liver cells HepG2 and mouse fibroblasts NIH-3T3, either 6,000 or 3,000 cells were seeded into each well of a 96-well plate, respectively. After 24 h, medium was supplemented with different concentrations of sodium arsenite (Sigma-Aldrich, order number 71287) and incubated for additional 24 h. After the incubation period, cells were washed once with PBS and subsequently lysed by contiguously shaking for 1 h at 4 °C in the presence of 35 μL NaOH (0.1 m) per well. Protein content was quantified with bicinchoninic acid assay kit (BCA Protein Assay, Thermo Fischer Scientific, Rockford, USA) according to the manufacturer's instructions.

### *C. elegans* experiments/maintenance and compound treatment

Nematode strains used in this study were provided by the Caenorhabditis Genetics Center (Univ. of Minnesota, USA). The following strains were used in this study: N2 wild-type (Bristol), *skn-1(zu67)*, *daf-16(mgDf47)*, *tin-9.1(ok2194)*, *mtl-2(gk125),* and *mev-1(kn1)*. Treatment of *C. elegans* was carried out on NGM agar plates containing the respective compound. Thereby, agar plates were prepared from the same batch of NGM Agar, whereas treatment plates were supplemented with the compound and control plates with the corresponding solvent as described (Schmeisser *et al*., 2011). After plates were poured and dried for about 30 min, they were sealed and stored at 4 °C.

Sodium arsenite treatment was performed using heat-inactivated bacteria (OP50 HIT) to avoid interference by the xenobiotic metabolizing activity of *E. coli* (Gruber *et al*., 2009). To obtain heat-inactivated bacteria, an overnight liquid culture of *E. coli* was treated 30 min at 65 °C. The bacteria suspension was then concentrated 20-fold by centrifugation (30 min at 3200 × g) and resuspended within S-buffer containing 10 mm MgSO_4_ and 5 μg mL^−1^ cholesterol as described (Wood *et al*., 2004). Furthermore, treatment NGM plates were prepared with 100 μg mL^−1^ ampicillin. It should be noted that this treatment causes an inactivation of metabolic activity of *E. coli* rather than a complete lethality. The antioxidants butylated hydroxyanisole (BHA) and *N*-acetyl-*L*-cysteine (NAC) were applied at a final concentration of 10 μm and 1 mm, respectively, as previously described (Zarse *et al*., 2012).

### *C. elegans* experiments/lifespan assays

All lifespan assays were performed at 20 °C according to standard protocols and as previously described (Schulz *et al*., 2007). Briefly, a *C. elegans* population was treated with hypochlorite/NaOH solution to synchronize the population at day zero of the lifespan. Eggs were transferred to freshly spotted plates to allow hatching and development. Soon after L4, around 150 nematodes were manually transferred to fresh incubation plates containing the respective compound or the solvent as control and spotted with a lawn of heat-inactivated OP50 *E. coli*.

For the first 10–14 days, worms were transferred every day and afterward 3–4 times a week. Nematodes that show no reaction to gently stimulation were scored as death. Those animals that crawled off the plates or display a premature, non-natural death due to internal hatching or bursting were censored (i.e., excluded from statistics on day of premature death).

### *C. elegans* experiments/locomotion

Single worm movements within a liquid system were recorded using a digital CCD camera (Moticam 2300, Motic, St. Ingbert, Germany) coupled to a microscope (SMZ 168, Motic, St. Ingbert, Germany) with a subsequent analysis using the program DanioTrack (Loligo Systems, Tjele, Denmark). Worms were transferred from agar plates to a buffer solution (S-buffer) and immediately afterward a 20-s video sequence was recorded. During the subsequent video analysis, the DanioTrack software subtracted the background and determined the center of gravity of all object pixels in contrast with the background. Finally, the moving distance of this worm gravity center was tracked and calculated, whereas the overall moving distance mainly refers to the body bends of the nematodes. It should be noted that worms that were transferred from their normal habitat (agar plated) to liquid respond with intensive body bends during the entire recording time. Thus, this locomotion assay can be considered as a quantitative analysis of the maximum movement capacity of a single worm. Moreover, in comparison with worm tracking on agar plates, the analysis under liquid conditions is free from special movement characteristics like crawling back and forth or complete movement stop, providing high level comparability.

### *C. elegans* experiments/thermotolerance

Thermotolerance was determined by measuring survival of worms exposed to lethal thermal stress (37 °C) using SYTOX® Green (Invitrogen, Carlsbad, CA, USA) and an automated single worm fluorescence progression determination on 384-well plates as previously described (Kampkötter *et al*., 2007). A detailed description can be found in the supplements.

### *C. elegans* experiments/paraquat stress resistance

Resistance to lethal oxidative stress derived from paraquat was determined with minor modifications as previously described (Schulz *et al*., 2007). Briefly, nematodes were pretreated with arsenite for 6 days. Thereafter, worms were transferred manually to fresh NGM plates containing 10 mm paraquat (Sigma-Aldrich) spotted with a lawn of heat-inactivated OP50 following by daily determining the survival rate until all nematodes were death. As described for lifespan analysis, worms were counted as censored in case of internal hatching, crawling off, and bursting.

### *C. elegans* experiments/mitochondrial reactive oxygen species quantification

To quantify mitochondrial ROS (mtROS) production, we used a commercial available dye, which enters the mitochondria in a reduced state (MitoTracker Red CM-H_2_X ROS; Invitrogen, Carlsbad, CA, USA). While the reduced probe does not exhibit any fluorescence, endogenous oxidation due to mitochondrial ROS production results in a detectable fluorescence active dye that accumulates in active mitochondria. To elucidate mtROS production in *C. elegans*, worms were treated with arsenite according to the descriptions above. Following that incubation period, nematodes were shifted to plates spotted with a mixture of dead OP50 (30 min at 65 °C in a water bath) and dye (final concentration 0.5 μm). Worms were incubated with the bacteria/dye mixture for 2 h with a subsequent 1-h recovery period on fresh plates (w/o dye) to eliminate artificial results due to large amounts of intestinal dye. Worms were washed at least 3 times when transferred to new plates. Finally, nematodes were distributed into wells of a FLUOTRAC™96-Well plate (Greiner Bio-One, Frickenhausen, Germany) and fluorescence was determined using a Fluorometer (FLUOstar Optima, BMG, Offenburg, Germany; Ex: 570 nm, Em: 610 nm; well-scanning mode). To normalize the fluorescence signal after measurement, worms were recovered from the wells and protein was analyzed as given above.

### *C. elegans* experiments/fluorescence microscopy

Worms were treated with MitoTracker Red CM-H_2_X ROS exactly as described for mtROS quantification. Individual worms were placed on agarose pads and paralyzed with tetramisole. Worms were examined under a fluorescence microscope (Axiovert 100, Zeiss, Oberkochen, Germany) using a specific filter set (BP546/12, FT580, LP590), and pictures were taken with a digital camera.

### *C. elegans* experiments/amplex red-based quantification of supernatant hydrogen peroxide

Worms were removed from plates with 0.05 m sodium phosphate buffer pH 7.4, washed twice, and transferred into an upright plexiglas cylinder (1.5 mL volume) with continuous stirring at low speed (20 rpm). Firstly, determination of fluorescence was carried out without horse radish peroxidase (HRP) (Sigma-Aldrich, St. Louis, MO, USA) only in the presence of 1 μm Amplex Red (Invitrogen, Carlsbad, CA, USA) to exclude a possible unspecific increase in fluorescence. Next, 0.01 U mL^−1^ HRP was added and changes of fluorescence were recorded with a fluorescence detector (LF402 ProLine, IOM, Berlin, Germany) for at least 15 min at excitation/emission wavelength of 571 nm and 585 nm, respectively. Immediately afterward, worms were removed and collected for protein determination to normalize fluorescence values.

### *C. elegans* experiments/catalase activity

Arsenite treated worms were harvested, washed with ice-cold buffer and disrupted by grinding (under liquid nitrogen together with 200 μL 50 mm phosphate buffer + 1 mm EDTA) and additional sonicating (3 times). The remaining lysate was cleared by centrifugation for 15 min at 12 000 × g and 4 °C. The supernatant was used for the subsequent measurement of catalase activity as well as for a protein determination (for normalizing catalase activity). Catalase activity quantification is based on the peroxidase activity of the enzyme, thus reacting with the hydrogen donor methanol in the presence of hydrogen peroxide to formaldehyde. Formaldehyde further reacts with the chromogen Purpald (4-amino-3-hydrazino-5-mercapto-1,2,4-triazole), leading to a bicyclic heterocycle complex that change the color upon potassium peroxide mediated oxidation from colorless to purple. The measurement was carried out like previously described with minor changes (Johansson & Borg, 1988). Briefly, 20 μL of the diluted (25 mm potassium phosphate buffer + 1 mm EDTA + 0.1% BSA, pH 7.5) sample supernatant was mixed with 100 μL assay buffer (100 mm potassium phosphate buffer, pH 7) and 30 μL methanol (VWR, Darmstadt, Germany). Next, 20 μL hydrogen peroxide (Applichem, Darmstadt, Germany, 30%) was added to initiate the reaction. After 20-min incubation under continuous shaking at 20 °C, 30 μL potassium hydroxide (Applichem, Darmstadt, Germany; 10 m) and 30 μL Purpald (Sigma-Aldrich, St. Louis, MO, USA; 46 mm in 0.5 m hydrochloric acid) were added to terminate the reaction. After a 10-min incubation time under continuous shaking at 20 °C, 10 μL potassium periodate (Sigma-Aldrich, St. Louis, MO, USA; 192 mm in 0.5 m potassium hydroxide) was added to oxidize the Purpald–formaldehyde complex and incubated for further 5 min. Interfering bubbles were removed by centrifugation. Finally, the absorbance was measured at 540 nm.

### *C. elegans* experiments/activity of superoxide dismutase

SOD activity was quantified like described (Peskin & Winterbourn, 2000) with minor changes: In general, the assay is based on the ability of SOD to prevent xanthine/xanthine oxidase mediated oxidation of WST-1 (2-(4-Iodophenyl)- 3-(4-nitrophenyl)-5-(2,4-disulfophenyl)- 2H-tetrazolium, monosodium salt; Applichem, Darmstadt, Germany). WST-1 produces a detectable formazan dye upon superoxide-mediated reduction; thus, SOD activity reduces the formation of the dye (Ishiyama *et al*., 1993). To quantify enzyme activity in worms, we firstly prepared nematodes as mentioned for catalase activity. SOD activity was determined in the remaining cleared and diluted worm lysate. Therefore, 200 μL WST-1 working solution (Tris–HCL pH 8, diethylenetriamine-pentaacetic acid (DTPA, Sigma-Aldrich, St. Louis, MO, USA, 100 μm), hypoxanthine (Applichem, Darmstadt, Germany, 100 μm), WST-1 (180 μm) was mixed with 20 μL sample and 20 μL xanthine oxidase (Sigma-Aldrich, St. Louis, MO, USA; 240 mU mL^−1^), following by a 20-min incubation period at 37 °C. Thereafter, absorbance was measured at 450 nm. For normalization of SOD activity, protein content of the sample was determined.

### *C. elegans* experiments/determination of reduced and oxidized glutathione

Determination of GSH and GSSG was carried out as described (Schulz *et al*., 2010). Treated worm samples were harvested, washed, and disrupted by grinding. The remaining powder was mixed with 0.8 mL chilled precipitating agent (acetonitrile and 10 mm KH2PO4; 3:1, v:v) and sonicated 3 times on ice. Precipitated proteins were separated by centrifugation (12 000 × g, 10 min 4 °C) and redissolved in sodium hydroxide solution for a subsequent protein determination. For the extraction of the metabolite-containing fraction, the supernatant was transferred into a new reaction tube and mixed with 2 mL chloroform. Samples were centrifuged 10 min at 3200 × g and 4 °C. The upper aqueous phase was aspirated and mixed with fresh chloroform. After an additional washing step, samples were subjected to HPLC separation (reverse-phase ion-pair HPLC; Jasco MD-1510; Gross-Umstadt, Germany) and detection (diode array detector; at 208 nm). Metabolites were identified by spiking of samples with appropriate standards. Metabolite content was normalized to protein content.

### *C. elegans* experiments/oxygen consumption

Respiration was quantified using a DW1/AD Clark-type electrode (Hansatech, King's Lynn, England) as previously described (Schulz *et al*., 2007). After individual incubation period, worms were harvested, washed and transferred into the DW1 chamber. Oxygen consumption was monitored for at least 10 min. Afterward, worms were carefully removed from the chamber and collected for a subsequent protein determination. Therefore, worms were sonicated 3 times and centrifuged for 10 min at 12 000 × g. Supernatant was used for protein determination using BCA, as described previously.

### *C. elegans* experiments/deep sequencing-based RNA expression analysis

For library preparation, an amount of 5 μg of total RNA per sample was processed using Illumina's mRNA-Seq sample prep kit (Illumina; San Diego; CA, USA) following the manufacturer's instruction. The libraries were sequenced using a Genome Analyser (GAIIx, Illumina, San Diego, CA, USA) in a single read approach with 76 cycles resulting in reads with length of 76 nucleotides. Each library was sequenced on a single lane ends up with around 30–40 mio reads per sample. Sequence data were extracted in FastQ format and used for mapping approach. All total reads that passed the quality filtering were mapped against the *C. elegans* genome and an exon junction splice database using Bowtie (Langmead *et al*., 2009). Only uniquely mapped reads were used for counting. The RefSeq annotation was used to assign mapping positions to exons, transcripts, and genes. The deep sequencing data obtained and discussed in the current publication have been deposited in NCBI's Gene Expression Omnibus and are accessible through GEO Series accession number GSE46257 (http://www.ncbi.nlm.nih.gov/geo/query/acc.cgi?acc=GSE46257).

### *C. elegans* experiments/promoter analyses

The search for SKN-1 and DAF-16 transcription factor binding sites (TFBS) was carried out within the proximal promoter region of all identified differential expressed genes 1.5 kb upstream of each predicted start codon.

### *C. elegans* experiments/gene ontology analyses

To classify the differential expressed genes, we used the database for annotation, visualization, and integrated discovery (DAVID) (Huang *et al*., 2009).

### All experiments/statistical analyses

Statistical analyses for all data except lifespan and stress resistance assays in *C. elegans* were performed by Student's *t*-test (unpaired, two-tailed) after testing for equal distribution of the data and equal variances within the data set. For comparing significant distributions between different groups in the lifespan assays and stress resistance assays, statistical calculations were carried out using the log-rank test. All calculations were performed using Excel 2007 (Microsoft, Albuquerque, NM, USA) and spss version 13.0 (IBM, Armonk, NY, USA). *P* < 0.05 was considered as statistically significant.

## Supporting Information

**Fig. S1** Individual results for N2 lifespan and paraquat stress resistance assays.

**Fig. S2** While arsenite accumulates within worms, low-dose arsenite exposure does not change food uptake and does not induce aversion behavior.

**Fig. S3** Like N2 nematodes exposed to low-dose arsenite, *mev-1* mutants display increased ROS formation but to a considerably larger extent.

**Fig. S4** *In silico* promoter analyses of differentially expressed genes.

**Table S1** Differentially expressed RNAs after 48 h of exposure to arsenite.

**Table S2** Functional classification of upregulated DEGs (FunCat).

**Table S3** Functional classification of upregulated DEGs (GO Term: Biological Process).

**Data S1** Supporting experimental procedures.
