# Supplementary material for: Mitochondrial hormesis links low-dose arsenite exposure to lifespan extension
Source: Aging Cell. 2013 May 6;12(3):508–17. doi: 10.1111/acel.12076 (PMC3709120; doi:10.1111/acel.12076)
Supplement: Supplementary file 1 [file acel0012-0508-SD1.doc]

**Supporting Information** to

**Mitochondrial Hormesis Links Low-Dose Arsenite Exposure to Lifespan Extension**

Sebastian Schmeisser, Kathrin Schmeisser, Sandra Weimer, Marco Groth, Steffen Priebe, Eugen Fazius, Doreen Kuhlow, Dennis Pick, Jürgen W. Einax, Reinhard Guthke, Matthias Platzer, Kim Zarse, Michael Ristow

**Suppl. Figure 1: Individual results for N2 lifespan and paraquat stress resistance assays** (page 2)

**Suppl. Figure 2: While arsenite accumulates within worms, low-dose arsenite exposure does not change food uptake and does not induce aversion behavior** (page 3)

**Suppl. Figure 3: Like N2 nematodes exposed to low-dose arsenite, *mev-1* mutants display increased ROS formation but to a considerably larger extent** (page 4)

**Suppl. Figure 4: *In silico* promoter analyses of differentially expressed genes** (page 5)

**Supporting experimental procedures** (pages 6-7)

**References to supporting experimental procedures** (page 8)

**Suppl. Table 1: Differentially expressed RNAs after 48 hours of exposure to arsenite** (page 9-15)

**Suppl. Table 2. Functional classification of upregulated DEGs (FunCat)** (page 16)

**Suppl. Table 3. Functional classification of upregulated DEGs (GO Term: Biological Process)** (page 17)

**Suppl. Fig. 1**

**(A)**

**(B)**

**(C)**

**(D)**

**(E)**

**(F)**

**(G)**

**(H)**

**(I)**

**Suppl. Fig. 1: Individual results for N2 lifespan and paraquat stress resistance assays.** (A to F) Individual lifespan data for wild-type worms in the absence (blue) and in the presence of 100 nM arsenite (red). (G to I) Individual paraquat stress resistance data depicting survival of untreated (blue) and arsenite pretreated (red) nematodes following exposure to paraquat stress.

**Suppl. Fig. 2**

**
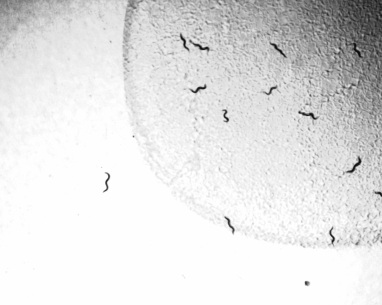

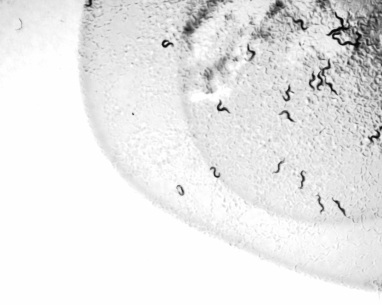

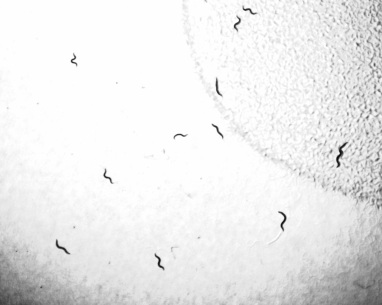
**

**Ctrl**

**AsO_2_^-^ 100nM**

**PQ 1mM**

**(B)**

**(C)**

**(D)**

**(A)**

**α**

**β**

**γ**

**α**

**β**

**Suppl. Fig. 2: While arsenite accumulates within worms, low-dose arsenite exposure does not change food uptake and does not induce aversion behaviour.** (A) Arsenic quantification in nematodes that have been exposed to different concentration of arsenite. (α) Summary of all concentrations, (β) exposure to 100 nM arsenite exclusively depicted. (B) Food uptake following exposure to arsenite for different time periods (blue: control wild-type worms, red: wild-type worms exposed to 100 nM arsenite). (C) Toxin-mediated aversion appearance for (α) solvent control, (β) 100 nM arsenite and (γ) 1 mM paraquat exposed animals. (D) Toxin-mediated aversion data for 3 independent experiments.

**Suppl. Fig. 3**

**(A)**

**(B)**

**Suppl. Fig. 3: Like N2 nematodes exposed to low-dose arsenite, *mev-1* mutants display increased ROS formation but to a considerably larger extent.** (A) Mitochondrial ROS formation following exposure to arsenite for different time periods (blue: control wild-type worms, red: wild-type worms exposed to 100 nM arsenite, grey: *C. elegans* *mev-1(kn1)* mutant, known to produce high levels of ROS, positive control). (B) Formation of hydrogen peroxide in nematode media following exposure to arsenite for different time periods (blue: control wild-type worms, red: wild-type worms exposed to 100 nM arsenite, grey: *C. elegans* *mev-1(kn-1)* mutant).

**Suppl. Fig. 4**

**(A)**


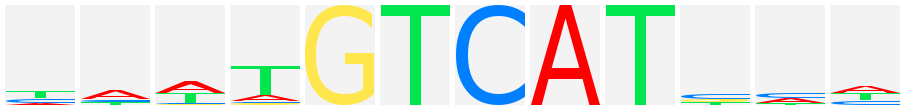


**(B)**


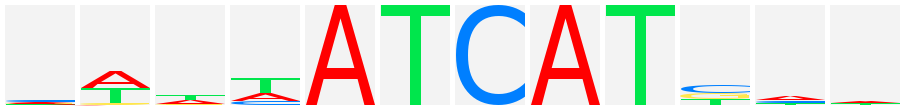


**(C)**


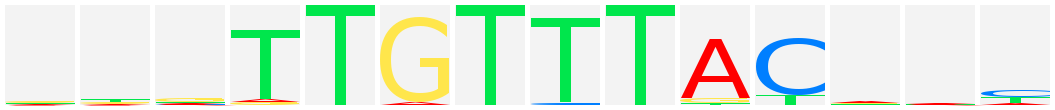


**(D)**


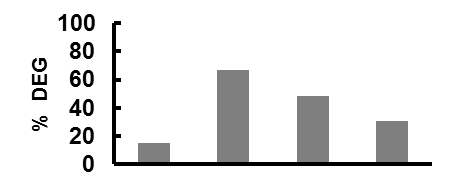


**neither SKN-1**

**nor DAF-16**

**SKN-1**

**DAF-16**

**both**

**Suppl. Fig. 4: *In silico* promoter analyses of differentially expressed genes.** (A and B) Depict the consensus sequences for the transcription factor SKN-1 and (C) DAF-16 our promoter analyses are based on and as previously published. (D) Results of *in silico* analyses of promoter consensus sequences for SKN-1, DAF-16, or both, in relative terms.

**Supporting experimental procedures**

***C. elegans* experiments / Thermotolerance:** The thermotolerance assay was performed as previously described . Briefly, the assay is based on a semi-automated determination of survival of worms exposed to lethal thermal stress (37°C) using SYTOX® Green (Invitrogen, Carlsbad, CA, USA) and a single worm fluorescence progression determination. Under normal conditions, SYTOX® Green is non-permeable to cell membranes. However, upon impairment of cell membranes, the dye can enter the cell and binds subsequently to the DNA. This causes a detectable change in fluorescence emission, that can be used as a marker for cellular damages and consequently for the vitality of the worms . For experiments, nematodes were treated with the respective compound for 6 days starting at L4 stage on NGM agar containing the compound and spotted with heat-inactivated bacteria. To avoid mixing of the population, worms were washed and transferred to fresh plates every day, whereas adult worms were separated from larvae and eggs by removing the supernatant several times after gravity-based separation. Thereafter, worms were harvested, washed and distributed individually to a 384-well plate (single worm per well). SYTOX® Green solved in S-Medium (1 µM final concentration) and OP50 (~10^7^/ml) were added to a final volume of 20 µl. Fluorescence intensity was measured every 15 minute using a Fluorometer (FLUOstar Optima, BMG, Offenburg, Germany; Ex: 485 nm, Em: 520 nm). Worms were defined as dead when the individual fluorescence of the corresponding worm exceeds a definite cut off value, which was calculated by multiplying the average fluorescence of the first four measurements by a factor of three.

***C. elegans* experiments / Extraction of total RNA:** RNA isolation was performed using a commercially available kit (Qiagen, Hilden, Germany, Rneasy Mini Kit) based on the phenol-chloroform extraction method according to the manufacturer’s instructions.

***C. elegans* experiments / Bioinformatical analysis of RNA expression data:** Raw counts for the transcripts were analyzed using the R Statistical Computing Environment and the Bioconductor package EdgeR . EdgeR provides statistical routines for determining differential expression in digital gene expression data using a model based on the negative binomial distribution. The resulting p-values were adjusted using the Benjamini and Hochberg's approach for controlling the false discovery rate (FDR) . Transcripts with an adjusted p-value smaller 0.05 were assigned as differentially expressed.

***C. elegans* experiments / Promoter analyses:** The search for SKN-1 and DAF-16 transcription factor binding sites (TFBS) was done within the proximal promoter region of all identified differential expressed genes 1.5 kb upstream of each predicted start codon. Therefore, a FASTA file containing all promoter regions of the corresponding differential expressed genes was created using WormMart . Next, the remaining sequence file was scanned for one or more matches to the position-specific scoring matrix (PSSM) of SKN-1 and DAF-16 using the matrix scan function of the pattern-matching program RSAT (regulatory sequence analysis tools) . The PSSM contains the nucleotide frequency at each position within the binding sites and were obtained for the database Transfac and experimentally verified as described . The threshold p-value, which indicates the risk of false positive predictions, was set to 0.0005.

***C. elegans* experiments / Arsenic quantification:** To quantify the amount of arsenic in worms we used dynamic reaction cell inductively coupled plasma mass spectrometry (ICP-DRC-MS) as previously described . Therefore, a high worm amount was collected subsequently to a 48h incubation period using different concentrations of arsenite. Worms were harvested and carefully washed several times to remove any arsenite containing bacteria and to allow excretion of the remaining intestinal content. After centrifugation, the whole supernatant was removed and the residual pellet was flash frozen in liquid nitrogen. The frozen material was weighed into a quartz digestion vessels and lysis solution was added (5 ml nitric acid and 2 ml hydrogen peroxide). Samples were incubated for 10 min and replaced into a microwave (5 min 100 W, 15 min 800 W). The homogenous solution was then transferred into 15 ml tubes, filled up to 15 ml total volume and measured using ICP-DRC-MS.

***C. elegans* experiments / Food uptake quantification:** To analyse the total amount of incorporated food, i.e. OP50 *E. coli*, nematodes were pretreated under standard conditions in the presence and absence of 100nM arsenite. Next, worms were transferred to assay plates that were spotted with a defined volume of heat inactivated OP50. Worms were allowed to consume bacteria for 6 hrs. Afterwards, the remaining bacteria and worms were thoroughly removed and transferred in a reaction tube. Worms were spun down at low speed and an aliquot of the supernatant was removed for a subsequent optical density (OD) determination using a microplate reader (FLUOstar Optima, BMG Labtech, Offenburg, Germany, at λ=600nm). An empty reference plate, i.e. w/o worms but with the same amount of bacteria, was equally handled. Δ OD was calculated by subtracting the OD of a worm plate by the OD value derived from the reference plate. The remaining worms were used for protein determination in order to normalize the OD 600 values.

***C. elegans* experiments / Toxin stimulated microbial aversions behaviour:** To analyse a potential influence of arsenite on microbial preference, we scored for aversion as described previously . The final concentration referred to the whole agar volume was 100 nM for arsenite and 1 mM for paraquat. Aversion was monitored every second hour (up to 8 hours in total).

**References to supporting experimental procedures**

Benjamini Y , Hochberg Y (1995). Controlling the false discovery rate: A practical and powerful approach to multiple testing. *J R Statist Soc B*. **57**, 289-300.

Blackwell TK, Bowerman B, Priess JR , Weintraub H (1994). Formation of a monomeric DNA binding domain by Skn-1 bZIP and homeodomain elements. *Science*. **266**, 621-628.

Furuyama T, Nakazawa T, Nakano I , Mori N (2000). Identification of the differential distribution patterns of mRNAs and consensus binding sequences for mouse DAF-16 homologues. *Biochem J*. **349**, 629-634.

Gill MS, Olsen A, Sampayo JN , Lithgow GJ (2003). An automated high-throughput assay for survival of the nematode Caenorhabditis elegans. *Free Radic Biol Med*. **35**, 558-565.

Kampkotter A, Gombitang Nkwonkam C, Zurawski RF, Timpel C, Chovolou Y, Watjen W , Kahl R (2007). Effects of the flavonoids kaempferol and fisetin on thermotolerance, oxidative stress and FoxO transcription factor DAF-16 in the model organism Caenorhabditis elegans. *Arch Toxicol*. **81**, 849-858.

Melo JA , Ruvkun G (2012). Inactivation of Conserved C. elegans Genes Engages Pathogen- and Xenobiotic-Associated Defenses. *Cell*. **149**, 452-466.

Pick D, Leiterer M , Einax JW (2010). Reduction of polyatomic interferences in biological material using dynamic reaction cell ICP-MS. *Microchem J*. **95**, 315-319.

Robinson MD, McCarthy DJ , Smyth GK (2010). edgeR: a Bioconductor package for differential expression analysis of digital gene expression data. *Bioinformatics*. **26**, 139-140.

Schwarz EM, Antoshechkin I, Bastiani C, Bieri T, Blasiar D, Canaran P, Chan J, Chen N, Chen WJ, Davis P, Fiedler TJ, Girard L, Harris TW, Kenny EE, Kishore R, Lawson D, Lee R, Muller HM, Nakamura C, Ozersky P, Petcherski A, Rogers A, Spooner W, Tuli MA, Van Auken K, Wang D, Durbin R, Spieth J, Stein LD , Sternberg PW (2006). WormBase: better software, richer content. *Nucleic Acids Res*. **34**, D475-478.

R Development Core Team (2008). *R: A language and environment for statistical computing*. Vienna.

Turatsinze JV, Thomas-Chollier M, Defrance M , van Helden J (2008). Using RSAT to scan genome sequences for transcription factor binding sites and cis-regulatory modules. *Nat Protoc*. **3**, 1578-1588.

Wingender E (2008). The TRANSFAC project as an example of framework technology that supports the analysis of genomic regulation. *Brief Bioinform*. **9**, 326-332.

**Suppl. Table 1: Differentially expressed RNAs after 48 hours of exposure to arsenite**

| **Gene Name** | **Description** | **Gene WB ID** | **Fold Change (log2)** | **P-Value (edgeR)** |
| --- | --- | --- | --- | --- |
|  |  |  |  |  |
| **F59B2.12** | hypothetical protein | [WBGene00010314](http://www.wormbase.org/db/gene/gene?name=WBGene00010314;class=Gene) | 4.09 | 0.00000 |
| **C14F11.7** | hypothetical protein | [WBGene00015782](http://www.wormbase.org/db/gene/gene?name=WBGene00015782;class=Gene) | 2.24 | 0.00882 |
| **Y59E9AR.1** | hypothetical protein | [WBGene00021997](http://www.wormbase.org/db/gene/gene?name=WBGene00021997;class=Gene) | 1.71 | 0.02554 |
| **Y59E9AR.7** | hypothetical protein | [WBGene00022002](http://www.wormbase.org/db/gene/gene?name=WBGene00022002;class=Gene) | 1.71 | 0.02554 |
| **F59C6.16** | hypothetical protein | [WBGene00077764](http://www.wormbase.org/db/gene/gene?name=WBGene00077764;class=Gene) | 1.70 | 0.00526 |
| **rpl-39** | Ribosomal Protein. Large subunit family member | [WBGene00004453](http://www.wormbase.org/db/gene/gene?name=WBGene00004453;class=Gene) | 1.56 | 0.00000 |
| **B0205.12** | hypothetical protein | [WBGene00044413](http://www.wormbase.org/db/gene/gene?name=WBGene00044413;class=Gene) | 1.49 | 0.00098 |
| **T07A5.5** | hypothetical protein | [WBGene00011558](http://www.wormbase.org/db/gene/gene?name=WBGene00011558;class=Gene) | 1.46 | 0.00000 |
| **F23A7.8** | hypothetical protein | [WBGene00044638](http://www.wormbase.org/db/gene/gene?name=WBGene00044638;class=Gene) | 1.45 | 0.00000 |
| **T23G11.11** | hypothetical protein | [WBGene00077728](http://www.wormbase.org/db/gene/gene?name=WBGene00077728;class=Gene) | 1.39 | 0.00226 |
| **F53A9.1** | hypothetical protein | [WBGene00018724](http://www.wormbase.org/db/gene/gene?name=WBGene00018724;class=Gene) | 1.37 | 0.00633 |
| **nlp-39** | Neuropeptide-Like Protein family member | [WBGene00008295](http://www.wormbase.org/db/gene/gene?name=WBGene00008295;class=Gene) | 1.34 | 0.01696 |
| **rps-28** | Ribosomal Protein. Small subunit family member | [WBGene00004497](http://www.wormbase.org/db/gene/gene?name=WBGene00004497;class=Gene) | 1.32 | 0.00000 |
| **T27A1.1** | hypothetical protein | [WBGene00005324](http://www.wormbase.org/db/gene/gene?name=WBGene00005324;class=Gene) | 1.24 | 0.00000 |
| **tomm-7** | Translocase of Outer Mitochondrial Membrane | [WBGene00022783](http://www.wormbase.org/db/gene/gene?name=WBGene00022783;class=Gene) | 1.20 | 0.00000 |
| **D1054.18** | hypothetical protein | [WBGene00077783](http://www.wormbase.org/db/gene/gene?name=WBGene00077783;class=Gene) | 1.19 | 0.03688 |
| **zig-7** | 2 (Zwei) IG-domain protein family member | [WBGene00006984](http://www.wormbase.org/db/gene/gene?name=WBGene00006984;class=Gene) | 1.18 | 0.00000 |
| **rpl-29** | Ribosomal Protein. Large subunit family member | [WBGene00004443](http://www.wormbase.org/db/gene/gene?name=WBGene00004443;class=Gene) | 1.16 | 0.00000 |
| **K08D9.1** | hypothetical protein | [WBGene00019523](http://www.wormbase.org/db/gene/gene?name=WBGene00019523;class=Gene) | 1.15 | 0.00001 |
| **Y22D7AR.10** | hypothetical protein | [WBGene00021263](http://www.wormbase.org/db/gene/gene?name=WBGene00021263;class=Gene) | 1.15 | 0.00000 |
| **nlp-30** | Neuropeptide-Like Protein family member | [WBGene00003768](http://www.wormbase.org/db/gene/gene?name=WBGene00003768;class=Gene) | 1.15 | 0.02366 |
| **C24H11.5** | hypothetical protein | [WBGene00007702](http://www.wormbase.org/db/gene/gene?name=WBGene00007702;class=Gene) | 1.14 | 0.00028 |
| **cnc-7** | CaeNaCin (Caenorhabditis bacteriocin) family | [WBGene00010005](http://www.wormbase.org/db/gene/gene?name=WBGene00010005;class=Gene) | 1.13 | 0.00119 |
| **Y38E10A.13** | hypothetical protein | [WBGene00012591](http://www.wormbase.org/db/gene/gene?name=WBGene00012591;class=Gene) | 1.13 | 0.01155 |
| **C08F8.9** | hypothetical protein | [WBGene00007449](http://www.wormbase.org/db/gene/gene?name=WBGene00007449;class=Gene) | 1.12 | 0.00000 |
| **Y39A3CL.3** | hypothetical protein | [WBGene00021441](http://www.wormbase.org/db/gene/gene?name=WBGene00021441;class=Gene) | 1.10 | 0.00000 |
| **C30G4.6** | hypothetical protein | [WBGene00016271](http://www.wormbase.org/db/gene/gene?name=WBGene00016271;class=Gene) | 1.09 | 0.00000 |
| **K06A4.7** | hypothetical protein | [WBGene00010597](http://www.wormbase.org/db/gene/gene?name=WBGene00010597;class=Gene) | 1.09 | 0.00000 |
| **Y51A2D.14** | hypothetical protein | [WBGene00013081](http://www.wormbase.org/db/gene/gene?name=WBGene00013081;class=Gene) | 1.09 | 0.00000 |
| **ZK512.4** | hypothetical protein | [WBGene00013984](http://www.wormbase.org/db/gene/gene?name=WBGene00013984;class=Gene) | 1.07 | 0.00000 |
| **F59C12.4** | hypothetical protein | [WBGene00045272](http://www.wormbase.org/db/gene/gene?name=WBGene00045272;class=Gene) | 1.06 | 0.00000 |
| **T07A9.15** | hypothetical protein | [WBGene00045249](http://www.wormbase.org/db/gene/gene?name=WBGene00045249;class=Gene) | 1.04 | 0.00098 |
| **C35C5.9** | hypothetical protein | [WBGene00007958](http://www.wormbase.org/db/gene/gene?name=WBGene00007958;class=Gene) | 1.01 | 0.01315 |
| **F12A10.1** | hypothetical protein | [WBGene00017390](http://www.wormbase.org/db/gene/gene?name=WBGene00017390;class=Gene) | 1.00 | 0.02032 |
| **Y37H9A.5** | hypothetical protein | [WBGene00014871](http://www.wormbase.org/db/gene/gene?name=WBGene00014871;class=Gene) | 0.99 | 0.00003 |
| **F15E6.4** | hypothetical protein | [WBGene00017485](http://www.wormbase.org/db/gene/gene?name=WBGene00017485;class=Gene) | 0.98 | 0.00112 |
| **nlp-28** | Neuropeptide-Like Protein family member | [WBGene00003766](http://www.wormbase.org/db/gene/gene?name=WBGene00003766;class=Gene) | 0.96 | 0.00000 |
| **F57B10.14** | hypothetical protein | [WBGene00019007](http://www.wormbase.org/db/gene/gene?name=WBGene00019007;class=Gene) | 0.94 | 0.00000 |
| **F35H10.5** | hypothetical protein | [WBGene00018070](http://www.wormbase.org/db/gene/gene?name=WBGene00018070;class=Gene) | 0.93 | 0.00066 |
| **F21C10.11** | hypothetical protein | [WBGene00017660](http://www.wormbase.org/db/gene/gene?name=WBGene00017660;class=Gene) | 0.90 | 0.02988 |
| **F25H5.8** | hypothetical protein | [WBGene00009130](http://www.wormbase.org/db/gene/gene?name=WBGene00009130;class=Gene) | 0.90 | 0.00706 |
| **grsp-1** | Glycine Rich Secreted Protein family member | [WBGene00012840](http://www.wormbase.org/db/gene/gene?name=WBGene00012840;class=Gene) | 0.90 | 0.00016 |
| **Y119D3B.21** | hypothetical protein | [WBGene00022497](http://www.wormbase.org/db/gene/gene?name=WBGene00022497;class=Gene) | 0.88 | 0.00000 |
| **F32D1.2** | hypothetical protein | [WBGene00017982](http://www.wormbase.org/db/gene/gene?name=WBGene00017982;class=Gene) | 0.87 | 0.00000 |
| **cnc-8** | CaeNaCin (Caenorhabditis bacteriocin) family | [WBGene00009624](http://www.wormbase.org/db/gene/gene?name=WBGene00009624;class=Gene) | 0.86 | 0.00008 |
| **F31E3.6** | hypothetical protein | [WBGene00017952](http://www.wormbase.org/db/gene/gene?name=WBGene00017952;class=Gene) | 0.85 | 0.00005 |
| **rps-29** | Ribosomal Protein. Small subunit family member | [WBGene00004498](http://www.wormbase.org/db/gene/gene?name=WBGene00004498;class=Gene) | 0.85 | 0.00000 |
| **Y38E10A.24** | hypothetical protein | [WBGene00012602](http://www.wormbase.org/db/gene/gene?name=WBGene00012602;class=Gene) | 0.82 | 0.00001 |
| **T09A12.5** | hypothetical protein | [WBGene00020374](http://www.wormbase.org/db/gene/gene?name=WBGene00020374;class=Gene) | 0.82 | 0.00000 |
| **F57C2.4** | hypothetical protein | [WBGene00010196](http://www.wormbase.org/db/gene/gene?name=WBGene00010196;class=Gene) | 0.81 | 0.00000 |
| **nlp-27** | Neuropeptide-Like Protein family member | [WBGene00003765](http://www.wormbase.org/db/gene/gene?name=WBGene00003765;class=Gene) | 0.77 | 0.00000 |
| **clp-1** | CaLPain family member (clp-1) | [WBGene00000542](http://www.wormbase.org/db/gene/gene?name=WBGene00000542;class=Gene) | 0.75 | 0.00005 |
| **C53H9.3** | hypothetical protein | [WBGene00044746](http://www.wormbase.org/db/gene/gene?name=WBGene00044746;class=Gene) | 0.75 | 0.00194 |
| **C10G8.4** | hypothetical protein | [WBGene00015683](http://www.wormbase.org/db/gene/gene?name=WBGene00015683;class=Gene) | 0.75 | 0.00000 |
| **rpb-12** | RNA Polymerase II (B) subunit family member | [WBGene00009078](http://www.wormbase.org/db/gene/gene?name=WBGene00009078;class=Gene) | 0.71 | 0.00036 |
| **B0495.6** | hypothetical protein | [WBGene00015205](http://www.wormbase.org/db/gene/gene?name=WBGene00015205;class=Gene) | 0.69 | 0.00036 |
| **T12B5.15** | hypothetical protein | [WBGene00050914](http://www.wormbase.org/db/gene/gene?name=WBGene00050914;class=Gene) | 0.69 | 0.00025 |
| **rpl-38** | Ribosomal Protein. Large subunit family member | [WBGene00004452](http://www.wormbase.org/db/gene/gene?name=WBGene00004452;class=Gene) | 0.69 | 0.00000 |
| **F23F1.10** | hypothetical protein | [WBGene00017750](http://www.wormbase.org/db/gene/gene?name=WBGene00017750;class=Gene) | 0.68 | 0.00406 |
| **nlp-33** | Neuropeptide-Like Protein family member | [WBGene00003771](http://www.wormbase.org/db/gene/gene?name=WBGene00003771;class=Gene) | 0.67 | 0.00013 |
| **nlp-31** | Neuropeptide-Like Protein family member | [WBGene00003769](http://www.wormbase.org/db/gene/gene?name=WBGene00003769;class=Gene) | 0.65 | 0.00013 |
| **rpl-43** | Ribosomal Protein. Large subunit family member | [WBGene00004456](http://www.wormbase.org/db/gene/gene?name=WBGene00004456;class=Gene) | 0.65 | 0.00000 |
| **F13G3.10** | hypothetical protein | [WBGene00008768](http://www.wormbase.org/db/gene/gene?name=WBGene00008768;class=Gene) | 0.65 | 0.00021 |
| **K10D2.4** | hypothetical protein | [WBGene00019630](http://www.wormbase.org/db/gene/gene?name=WBGene00019630;class=Gene) | 0.65 | 0.00968 |
| **snr-7** | Small Nuclear Ribonucleoprotein family member | [WBGene00004920](http://www.wormbase.org/db/gene/gene?name=WBGene00004920;class=Gene) | 0.64 | 0.00000 |
| **ubl-5** | UBiquitin-Like family member (ubl-5) | [WBGene00006726](http://www.wormbase.org/db/gene/gene?name=WBGene00006726;class=Gene) | 0.63 | 0.00392 |
| **mtl-2** | MeTaLlothionein family member (mtl-2) | [WBGene00003474](http://www.wormbase.org/db/gene/gene?name=WBGene00003474;class=Gene) | 0.62 | 0.00003 |
| **C14B9.10** | hypothetical protein | [WBGene00015755](http://www.wormbase.org/db/gene/gene?name=WBGene00015755;class=Gene) | 0.60 | 0.00002 |
| **K11H3.6** | hypothetical protein | [WBGene00010783](http://www.wormbase.org/db/gene/gene?name=WBGene00010783;class=Gene) | 0.59 | 0.01004 |
| **rps-21** | Ribosomal Protein. Small subunit family member | [WBGene00004490](http://www.wormbase.org/db/gene/gene?name=WBGene00004490;class=Gene) | 0.58 | 0.00001 |
| **ins-19** | INSulin related family member (ins-19) | [WBGene00002102](http://www.wormbase.org/db/gene/gene?name=WBGene00002102;class=Gene) | 0.58 | 0.03420 |
| **Y71H2B.4** | hypothetical protein | [WBGene00022194](http://www.wormbase.org/db/gene/gene?name=WBGene00022194;class=Gene) | 0.57 | 0.00281 |
| **nlp-29** | Neuropeptide-Like Protein family member | [WBGene00003767](http://www.wormbase.org/db/gene/gene?name=WBGene00003767;class=Gene) | 0.55 | 0.00038 |
| **F42H10.2** | hypothetical protein | [WBGene00018366](http://www.wormbase.org/db/gene/gene?name=WBGene00018366;class=Gene) | 0.53 | 0.01315 |
| **tin-9.1** | Transport to INner mitochondrial membrane (yeast | [WBGene00006572](http://www.wormbase.org/db/gene/gene?name=WBGene00006572;class=Gene) | 0.52 | 0.02072 |
| **B0513.4** | hypothetical protein | [WBGene00007196](http://www.wormbase.org/db/gene/gene?name=WBGene00007196;class=Gene) | 0.52 | 0.00038 |
| **spp-17** | SaPosin-like Protein family member (spp-17) | [WBGene00005002](http://www.wormbase.org/db/gene/gene?name=WBGene00005002;class=Gene) | 0.49 | 0.01764 |
| **rpb-10** | RNA Polymerase II (B) subunit family member | [WBGene00021347](http://www.wormbase.org/db/gene/gene?name=WBGene00021347;class=Gene) | 0.49 | 0.01012 |
| **R09B3.2** | hypothetical protein | [WBGene00011155](http://www.wormbase.org/db/gene/gene?name=WBGene00011155;class=Gene) | 0.48 | 0.00098 |
| **B0285.3** | hypothetical protein | [WBGene00007136](http://www.wormbase.org/db/gene/gene?name=WBGene00007136;class=Gene) | 0.48 | 0.01102 |
| **rps-27** | Ribosomal Protein. Small subunit family member | [WBGene00004496](http://www.wormbase.org/db/gene/gene?name=WBGene00004496;class=Gene) | 0.46 | 0.00138 |
| **F15D4.3** | hypothetical protein | [WBGene00008860](http://www.wormbase.org/db/gene/gene?name=WBGene00008860;class=Gene) | 0.46 | 0.01954 |
| **snr-6** | Small Nuclear Ribonucleoprotein family member | [WBGene00004919](http://www.wormbase.org/db/gene/gene?name=WBGene00004919;class=Gene) | 0.45 | 0.00706 |
| **kbp-4** | KNL (kinetochore null) Binding Protein family | [WBGene00022357](http://www.wormbase.org/db/gene/gene?name=WBGene00022357;class=Gene) | 0.43 | 0.04319 |
| **rpl-41** | Ribosomal Protein. Large subunit family member | [WBGene00004454](http://www.wormbase.org/db/gene/gene?name=WBGene00004454;class=Gene) | 0.39 | 0.01960 |
| **Y97E10C.1** | hypothetical protein | [WBGene00022410](http://www.wormbase.org/db/gene/gene?name=WBGene00022410;class=Gene) | -0.30 | 0.02855 |
| **glf-1** | GaLactoFuranose synthesis (UGM. | [WBGene00019154](http://www.wormbase.org/db/gene/gene?name=WBGene00019154;class=Gene) | -0.31 | 0.02661 |
| **T21B6.3** | hypothetical protein | [WBGene00011880](http://www.wormbase.org/db/gene/gene?name=WBGene00011880;class=Gene) | -0.31 | 0.02072 |
| **bus-8** | Bacterially Un-Swollen (M. nematophilum | [WBGene00044623](http://www.wormbase.org/db/gene/gene?name=WBGene00044623;class=Gene) | -0.32 | 0.02632 |
| **chs-2** | CHitin Synthase family member (chs-2) | [WBGene00000497](http://www.wormbase.org/db/gene/gene?name=WBGene00000497;class=Gene) | -0.32 | 0.02987 |
| **myo-2** | MYOsin heavy chain structural genes family | [WBGene00003514](http://www.wormbase.org/db/gene/gene?name=WBGene00003514;class=Gene) | -0.32 | 0.00815 |
| **ptr-18** | PaTched Related family member (ptr-18) | [WBGene00004232](http://www.wormbase.org/db/gene/gene?name=WBGene00004232;class=Gene) | -0.33 | 0.04052 |
| **slo-1** | SLOwpoke potassium channel family member | [WBGene00004830](http://www.wormbase.org/db/gene/gene?name=WBGene00004830;class=Gene) | -0.33 | 0.03433 |
| **ZC123.1** | hypothetical protein | [WBGene00022517](http://www.wormbase.org/db/gene/gene?name=WBGene00022517;class=Gene) | -0.33 | 0.02072 |
| **Y97E10AR.1** | hypothetical protein | [WBGene00022396](http://www.wormbase.org/db/gene/gene?name=WBGene00022396;class=Gene) | -0.34 | 0.01898 |
| **F33D4.6** | hypothetical protein | [WBGene00017998](http://www.wormbase.org/db/gene/gene?name=WBGene00017998;class=Gene) | -0.34 | 0.01608 |
| **F13H6.1** | hypothetical protein | [WBGene00017430](http://www.wormbase.org/db/gene/gene?name=WBGene00017430;class=Gene) | -0.35 | 0.01065 |
| **dpy-1** | DumPY : shorter than wild-type family member | [WBGene00001063](http://www.wormbase.org/db/gene/gene?name=WBGene00001063;class=Gene) | -0.35 | 0.02272 |
| **R11A5.7** | hypothetical protein | [WBGene00011235](http://www.wormbase.org/db/gene/gene?name=WBGene00011235;class=Gene) | -0.35 | 0.03580 |
| **ace-3** | abnormal ACEtylcholinesterase family member | [WBGene00000037](http://www.wormbase.org/db/gene/gene?name=WBGene00000037;class=Gene) | -0.36 | 0.02554 |
| **F22F4.1** | hypothetical protein | [WBGene00017716](http://www.wormbase.org/db/gene/gene?name=WBGene00017716;class=Gene) | -0.36 | 0.01714 |
| **unc-1** | UNCoordinated family member (unc-1) | [WBGene00006741](http://www.wormbase.org/db/gene/gene?name=WBGene00006741;class=Gene) | -0.36 | 0.04507 |
| **col-165** | COLlagen family member (col-165) | [WBGene00000738](http://www.wormbase.org/db/gene/gene?name=WBGene00000738;class=Gene) | -0.36 | 0.00302 |
| **wrt-1** | WaRThog (hedgehog-like family) family member | [WBGene00006947](http://www.wormbase.org/db/gene/gene?name=WBGene00006947;class=Gene) | -0.37 | 0.00340 |
| **unc-41** | UNCoordinated family member (unc-41) | [WBGene00006777](http://www.wormbase.org/db/gene/gene?name=WBGene00006777;class=Gene) | -0.37 | 0.01136 |
| **col-74** | COLlagen family member (col-74) | [WBGene00000650](http://www.wormbase.org/db/gene/gene?name=WBGene00000650;class=Gene) | -0.37 | 0.00180 |
| **ZK154.1** | hypothetical protein | [WBGene00022665](http://www.wormbase.org/db/gene/gene?name=WBGene00022665;class=Gene) | -0.37 | 0.03925 |
| **grl-7** | GRound-Like (grd related) family member (grl-7) | [WBGene00001716](http://www.wormbase.org/db/gene/gene?name=WBGene00001716;class=Gene) | -0.37 | 0.01315 |
| **pqn-46** | Prion-like-(Q/N-rich)-domain-bearing protein | [WBGene00004133](http://www.wormbase.org/db/gene/gene?name=WBGene00004133;class=Gene) | -0.38 | 0.00599 |
| **unc-25** | UNCoordinated family member (unc-25) | [WBGene00006762](http://www.wormbase.org/db/gene/gene?name=WBGene00006762;class=Gene) | -0.38 | 0.02305 |
| **grl-15** | GRound-Like (grd related) family member | [WBGene00001724](http://www.wormbase.org/db/gene/gene?name=WBGene00001724;class=Gene) | -0.38 | 0.04292 |
| **unc-9** | UNCoordinated family member (unc-9) | [WBGene00006749](http://www.wormbase.org/db/gene/gene?name=WBGene00006749;class=Gene) | -0.38 | 0.02272 |
| **nspc-17** | Nematode Specific Peptide family. group C family | [WBGene00008359](http://www.wormbase.org/db/gene/gene?name=WBGene00008359;class=Gene) | -0.39 | 0.02092 |
| **mlt-9** | MoLTing defective family member (mlt-9) | [WBGene00008605](http://www.wormbase.org/db/gene/gene?name=WBGene00008605;class=Gene) | -0.39 | 0.00214 |
| **feh-1** | mammalian FE65 Homolog family member (feh-1) | [WBGene00001410](http://www.wormbase.org/db/gene/gene?name=WBGene00001410;class=Gene) | -0.39 | 0.02235 |
| **F45G2.2** | hypothetical protein | [WBGene00009730](http://www.wormbase.org/db/gene/gene?name=WBGene00009730;class=Gene) | -0.40 | 0.00774 |
| **pqn-74** | Prion-like-(Q/N-rich)-domain-bearing protein | [WBGene00004156](http://www.wormbase.org/db/gene/gene?name=WBGene00004156;class=Gene) | -0.40 | 0.03495 |
| **peroxidase** | hypothetical protein | [WBGene00016700](http://www.wormbase.org/db/gene/gene?name=WBGene00016700;class=Gene) | -0.41 | 0.00469 |
| **chitinase** | hypothetical protein | [WBGene00007425](http://www.wormbase.org/db/gene/gene?name=WBGene00007425;class=Gene) | -0.41 | 0.04491 |
| **pqn-95** | Prion-like-(Q/N-rich)-domain-bearing protein | [WBGene00004174](http://www.wormbase.org/db/gene/gene?name=WBGene00004174;class=Gene) | -0.41 | 0.00062 |
| **asm-3** | Acid SphingoMyelinase family member (asm-3) | [WBGene00000213](http://www.wormbase.org/db/gene/gene?name=WBGene00000213;class=Gene) | -0.41 | 0.01260 |
| **F20D6.10** | hypothetical protein | [WBGene00017639](http://www.wormbase.org/db/gene/gene?name=WBGene00017639;class=Gene) | -0.41 | 0.04507 |
| **col-14** | COLlagen family member (col-14) | [WBGene00000603](http://www.wormbase.org/db/gene/gene?name=WBGene00000603;class=Gene) | -0.42 | 0.01679 |
| **F01G10.9** | hypothetical protein | [WBGene00008511](http://www.wormbase.org/db/gene/gene?name=WBGene00008511;class=Gene) | -0.42 | 0.04002 |
| **inx-6** | INneXin family member (inx-6) | [WBGene00002128](http://www.wormbase.org/db/gene/gene?name=WBGene00002128;class=Gene) | -0.42 | 0.01802 |
| **F13H8.5** | hypothetical protein | [WBGene00017438](http://www.wormbase.org/db/gene/gene?name=WBGene00017438;class=Gene) | -0.43 | 0.00938 |
| **cut-3** | CUTiclin family member (cut-3) | [WBGene00009041](http://www.wormbase.org/db/gene/gene?name=WBGene00009041;class=Gene) | -0.43 | 0.00011 |
| **ZK675.4** | hypothetical protein | [WBGene00014067](http://www.wormbase.org/db/gene/gene?name=WBGene00014067;class=Gene) | -0.43 | 0.04087 |
| **C49F8.3** | hypothetical protein | [WBGene00008215](http://www.wormbase.org/db/gene/gene?name=WBGene00008215;class=Gene) | -0.43 | 0.01836 |
| **mec-1** | MEChanosensory abnormality family member | [WBGene00003165](http://www.wormbase.org/db/gene/gene?name=WBGene00003165;class=Gene) | -0.44 | 0.03160 |
| **peroxidase** | hypothetical protein | [WBGene00011530](http://www.wormbase.org/db/gene/gene?name=WBGene00011530;class=Gene) | -0.44 | 0.00046 |
| **F45E4.3** | hypothetical protein | [WBGene00018468](http://www.wormbase.org/db/gene/gene?name=WBGene00018468;class=Gene) | -0.44 | 0.00232 |
| **phat-5** | PHAryngeal gland Toxin-related family member | [WBGene00020242](http://www.wormbase.org/db/gene/gene?name=WBGene00020242;class=Gene) | -0.44 | 0.02217 |
| **F53B1.4** | hypothetical protein | [WBGene00018737](http://www.wormbase.org/db/gene/gene?name=WBGene00018737;class=Gene) | -0.45 | 0.00067 |
| **F53F8.4** | hypothetical protein | [WBGene00010001](http://www.wormbase.org/db/gene/gene?name=WBGene00010001;class=Gene) | -0.45 | 0.04754 |
| **lgc-34** | Ligand-Gated ion Channel family member (lgc-34) | [WBGene00020836](http://www.wormbase.org/db/gene/gene?name=WBGene00020836;class=Gene) | -0.45 | 0.00602 |
| **qui-1** | QUInine non-avoider family member (qui-1) | [WBGene00004265](http://www.wormbase.org/db/gene/gene?name=WBGene00004265;class=Gene) | -0.46 | 0.01749 |
| **BE10.2** | hypothetical protein | [WBGene00007210](http://www.wormbase.org/db/gene/gene?name=WBGene00007210;class=Gene) | -0.46 | 0.00460 |
| **daf-6** | abnormal DAuer Formation family member (daf-6) | [WBGene00000902](http://www.wormbase.org/db/gene/gene?name=WBGene00000902;class=Gene) | -0.46 | 0.02072 |
| **olrn-1** | Olfactory LeaRNing defective family member | [WBGene00007260](http://www.wormbase.org/db/gene/gene?name=WBGene00007260;class=Gene) | -0.46 | 0.01663 |
| **F10D11.6** | hypothetical protein | [WBGene00008652](http://www.wormbase.org/db/gene/gene?name=WBGene00008652;class=Gene) | -0.47 | 0.00012 |
| **ZK84.1** | hypothetical protein | [WBGene00022649](http://www.wormbase.org/db/gene/gene?name=WBGene00022649;class=Gene) | -0.47 | 0.00208 |
| **F32D8.7** | hypothetical protein | [WBGene00009331](http://www.wormbase.org/db/gene/gene?name=WBGene00009331;class=Gene) | -0.47 | 0.02987 |
| **mpk-2** | MAP Kinase family member (mpk-2) | [WBGene00003402](http://www.wormbase.org/db/gene/gene?name=WBGene00003402;class=Gene) | -0.47 | 0.01328 |
| **col-104** | COLlagen family member (col-104) | [WBGene00000678](http://www.wormbase.org/db/gene/gene?name=WBGene00000678;class=Gene) | -0.47 | 0.03198 |
| **cuticlin** | hypothetical protein | [WBGene00009983](http://www.wormbase.org/db/gene/gene?name=WBGene00009983;class=Gene) | -0.47 | 0.00003 |
| **R74.2** | hypothetical protein | [WBGene00011278](http://www.wormbase.org/db/gene/gene?name=WBGene00011278;class=Gene) | -0.47 | 0.00541 |
| **clec-72** | C-type LECtin family member (clec-72) | [WBGene00021583](http://www.wormbase.org/db/gene/gene?name=WBGene00021583;class=Gene) | -0.48 | 0.01389 |
| **unc-17** | UNCoordinated family member (unc-17) | [WBGene00000481](http://www.wormbase.org/db/gene/gene?name=WBGene00000481;class=Gene) | -0.49 | 0.01878 |
| **T06A1.1** | hypothetical protein | [WBGene00020277](http://www.wormbase.org/db/gene/gene?name=WBGene00020277;class=Gene) | -0.50 | 0.04312 |
| **T27C4.2** | hypothetical protein | [WBGene00020855](http://www.wormbase.org/db/gene/gene?name=WBGene00020855;class=Gene) | -0.50 | 0.02955 |
| **grl-16** | GRound-Like (grd related) family member | [WBGene00001725](http://www.wormbase.org/db/gene/gene?name=WBGene00001725;class=Gene) | -0.50 | 0.00009 |
| **abu-8** | Activated in Blocked Unfolded protein response | [WBGene00000031](http://www.wormbase.org/db/gene/gene?name=WBGene00000031;class=Gene) | -0.51 | 0.02630 |
| **col-166** | COLlagen family member (col-166) | [WBGene00000739](http://www.wormbase.org/db/gene/gene?name=WBGene00000739;class=Gene) | -0.52 | 0.00002 |
| **ifp-1** | Intermediate Filament Protein. class E family | [WBGene00002067](http://www.wormbase.org/db/gene/gene?name=WBGene00002067;class=Gene) | -0.52 | 0.00001 |
| **col-10** | COLlagen family member (col-10) | [WBGene00000599](http://www.wormbase.org/db/gene/gene?name=WBGene00000599;class=Gene) | -0.53 | 0.00118 |
| **mec-2** | MEChanosensory abnormality family member | [WBGene00003166](http://www.wormbase.org/db/gene/gene?name=WBGene00003166;class=Gene) | -0.53 | 0.00695 |
| **F56F12.1** | hypothetical protein | [WBGene00010156](http://www.wormbase.org/db/gene/gene?name=WBGene00010156;class=Gene) | -0.53 | 0.00977 |
| **phat-2** | PHAryngeal gland Toxin-related family member | [WBGene00016733](http://www.wormbase.org/db/gene/gene?name=WBGene00016733;class=Gene) | -0.53 | 0.00428 |
| **cuticlin** | hypothetical protein | [WBGene00018297](http://www.wormbase.org/db/gene/gene?name=WBGene00018297;class=Gene) | -0.53 | 0.00020 |
| **col-130** | COLlagen family member (col-130) | [WBGene00000704](http://www.wormbase.org/db/gene/gene?name=WBGene00000704;class=Gene) | -0.53 | 0.03681 |
| **bus-19** | Bacterially Un-Swollen (M. nematophilum | [WBGene00011590](http://www.wormbase.org/db/gene/gene?name=WBGene00011590;class=Gene) | -0.53 | 0.00073 |
| **T19C3.2** | hypothetical protein | [WBGene00020560](http://www.wormbase.org/db/gene/gene?name=WBGene00020560;class=Gene) | -0.53 | 0.02524 |
| **glr-1** | GLutamate Receptor family (AMPA) family member | [WBGene00001612](http://www.wormbase.org/db/gene/gene?name=WBGene00001612;class=Gene) | -0.54 | 0.02615 |
| **ZK180.5** | hypothetical protein | [WBGene00022679](http://www.wormbase.org/db/gene/gene?name=WBGene00022679;class=Gene) | -0.54 | 0.00000 |
| **F14B6.2** | hypothetical protein | [WBGene00008783](http://www.wormbase.org/db/gene/gene?name=WBGene00008783;class=Gene) | -0.54 | 0.01371 |
| **abu-11** | Activated in Blocked Unfolded protein response | [WBGene00000034](http://www.wormbase.org/db/gene/gene?name=WBGene00000034;class=Gene) | -0.55 | 0.04531 |
| **ZC581.9** | hypothetical protein | [WBGene00022635](http://www.wormbase.org/db/gene/gene?name=WBGene00022635;class=Gene) | -0.55 | 0.03304 |
| **col-48** | COLlagen family member (col-48) | [WBGene00000625](http://www.wormbase.org/db/gene/gene?name=WBGene00000625;class=Gene) | -0.56 | 0.00047 |
| **carboxypeptidase** | hypothetical protein | [WBGene00020283](http://www.wormbase.org/db/gene/gene?name=WBGene00020283;class=Gene) | -0.57 | 0.00321 |
| **grl-5** | GRound-Like (grd related) family member (grl-5) | [WBGene00001714](http://www.wormbase.org/db/gene/gene?name=WBGene00001714;class=Gene) | -0.57 | 0.00133 |
| **Y53F4B.27** | hypothetical protein | [WBGene00013173](http://www.wormbase.org/db/gene/gene?name=WBGene00013173;class=Gene) | -0.58 | 0.00119 |
| **rol-6** | ROLler: helically twisted. animals roll when | [WBGene00004397](http://www.wormbase.org/db/gene/gene?name=WBGene00004397;class=Gene) | -0.58 | 0.00977 |
| **cuticlin** | hypothetical protein | [WBGene00009982](http://www.wormbase.org/db/gene/gene?name=WBGene00009982;class=Gene) | -0.59 | 0.00000 |
| **C34H4.5** | hypothetical protein | [WBGene00016427](http://www.wormbase.org/db/gene/gene?name=WBGene00016427;class=Gene) | -0.60 | 0.00000 |
| **C10F3.7** | hypothetical protein | [WBGene00015679](http://www.wormbase.org/db/gene/gene?name=WBGene00015679;class=Gene) | -0.61 | 0.00469 |
| **Y47D3B.6** | hypothetical protein | [WBGene00012942](http://www.wormbase.org/db/gene/gene?name=WBGene00012942;class=Gene) | -0.61 | 0.00073 |
| **unc-93** | UNCoordinated family member (unc-93) | [WBGene00006822](http://www.wormbase.org/db/gene/gene?name=WBGene00006822;class=Gene) | -0.62 | 0.03608 |
| **daf-11** | abnormal DAuer Formation family member (daf-11) | [WBGene00000907](http://www.wormbase.org/db/gene/gene?name=WBGene00000907;class=Gene) | -0.62 | 0.01878 |
| **col-65** | COLlagen family member (col-65) | [WBGene00000641](http://www.wormbase.org/db/gene/gene?name=WBGene00000641;class=Gene) | -0.62 | 0.00000 |
| **T25E4.1** | hypothetical protein | [WBGene00020803](http://www.wormbase.org/db/gene/gene?name=WBGene00020803;class=Gene) | -0.62 | 0.01819 |
| **ZC434.3** | hypothetical protein | [WBGene00013891](http://www.wormbase.org/db/gene/gene?name=WBGene00013891;class=Gene) | -0.63 | 0.01878 |
| **F30A10.2** | hypothetical protein | [WBGene00009261](http://www.wormbase.org/db/gene/gene?name=WBGene00009261;class=Gene) | -0.63 | 0.00032 |
| **col-39** | COLlagen family member (col-39) | [WBGene00000616](http://www.wormbase.org/db/gene/gene?name=WBGene00000616;class=Gene) | -0.63 | 0.00020 |
| **Y47D7A.13** | hypothetical protein | [WBGene00021625](http://www.wormbase.org/db/gene/gene?name=WBGene00021625;class=Gene) | -0.64 | 0.00000 |
| **tag-68** | Temporarily Assigned Gene name family member | [WBGene00006445](http://www.wormbase.org/db/gene/gene?name=WBGene00006445;class=Gene) | -0.64 | 0.01077 |
| **Y106G6G.2** | hypothetical protein | [WBGene00013711](http://www.wormbase.org/db/gene/gene?name=WBGene00013711;class=Gene) | -0.64 | 0.00706 |
| **col-97** | COLlagen family member (col-97) | [WBGene00000672](http://www.wormbase.org/db/gene/gene?name=WBGene00000672;class=Gene) | -0.65 | 0.00001 |
| **H10E21.4** | hypothetical protein | [WBGene00019184](http://www.wormbase.org/db/gene/gene?name=WBGene00019184;class=Gene) | -0.65 | 0.00045 |
| **lgc-30** | Ligand-Gated ion Channel family member (lgc-30) | [WBGene00019069](http://www.wormbase.org/db/gene/gene?name=WBGene00019069;class=Gene) | -0.65 | 0.02939 |
| **col-77** | COLlagen family member (col-77) | [WBGene00000653](http://www.wormbase.org/db/gene/gene?name=WBGene00000653;class=Gene) | -0.67 | 0.00001 |
| **col-125** | COLlagen family member (col-125) | [WBGene00000699](http://www.wormbase.org/db/gene/gene?name=WBGene00000699;class=Gene) | -0.67 | 0.00000 |
| **F59B10.3** | hypothetical protein | [WBGene00010319](http://www.wormbase.org/db/gene/gene?name=WBGene00010319;class=Gene) | -0.67 | 0.04919 |
| **Y95B8A.2** | hypothetical protein | [WBGene00022383](http://www.wormbase.org/db/gene/gene?name=WBGene00022383;class=Gene) | -0.69 | 0.00606 |
| **ZK829.3** | hypothetical protein | [WBGene00014094](http://www.wormbase.org/db/gene/gene?name=WBGene00014094;class=Gene) | -0.69 | 0.01742 |
| **T14A8.2** | hypothetical protein | [WBGene00020497](http://www.wormbase.org/db/gene/gene?name=WBGene00020497;class=Gene) | -0.70 | 0.00480 |
| **rol-8** | ROLler: helically twisted. animals roll when | [WBGene00004398](http://www.wormbase.org/db/gene/gene?name=WBGene00004398;class=Gene) | -0.70 | 0.00278 |
| **lgc-21** | Ligand-Gated ion Channel family member (lgc-21) | [WBGene00007903](http://www.wormbase.org/db/gene/gene?name=WBGene00007903;class=Gene) | -0.70 | 0.00022 |
| **T21C9.9** | hypothetical protein | [WBGene00011895](http://www.wormbase.org/db/gene/gene?name=WBGene00011895;class=Gene) | -0.71 | 0.01765 |
| **col-167** | COLlagen family member (col-167) | [WBGene00000740](http://www.wormbase.org/db/gene/gene?name=WBGene00000740;class=Gene) | -0.72 | 0.00000 |
| **F41E6.11** | hypothetical protein | [WBGene00018292](http://www.wormbase.org/db/gene/gene?name=WBGene00018292;class=Gene) | -0.72 | 0.04491 |
| **col-107** | COLlagen family member (col-107) | [WBGene00000681](http://www.wormbase.org/db/gene/gene?name=WBGene00000681;class=Gene) | -0.73 | 0.00000 |
| **phat-4** | PHAryngeal gland Toxin-related family member | [WBGene00020237](http://www.wormbase.org/db/gene/gene?name=WBGene00020237;class=Gene) | -0.74 | 0.00009 |
| **grd-13** | GRounDhog (hedgehog-like family) family member | [WBGene00001702](http://www.wormbase.org/db/gene/gene?name=WBGene00001702;class=Gene) | -0.74 | 0.00480 |
| **ZK180.6** | hypothetical protein | [WBGene00022680](http://www.wormbase.org/db/gene/gene?name=WBGene00022680;class=Gene) | -0.74 | 0.00253 |
| **ram-2** | abnormal RAy Morphology family member (ram-2) | [WBGene00004300](http://www.wormbase.org/db/gene/gene?name=WBGene00004300;class=Gene) | -0.74 | 0.00000 |
| **nas-38** | Nematode AStacin protease family member | [WBGene00003554](http://www.wormbase.org/db/gene/gene?name=WBGene00003554;class=Gene) | -0.74 | 0.04491 |
| **C05E7.1** | hypothetical protein | [WBGene00007343](http://www.wormbase.org/db/gene/gene?name=WBGene00007343;class=Gene) | -0.74 | 0.00075 |
| **grd-14** | GRounDhog (hedgehog-like family) family member | [WBGene00001703](http://www.wormbase.org/db/gene/gene?name=WBGene00001703;class=Gene) | -0.75 | 0.01368 |
| **clec-170** | C-type LECtin family member (clec-170) | [WBGene00009515](http://www.wormbase.org/db/gene/gene?name=WBGene00009515;class=Gene) | -0.75 | 0.03681 |
| **R05A10.1** | hypothetical protein | [WBGene00011018](http://www.wormbase.org/db/gene/gene?name=WBGene00011018;class=Gene) | -0.76 | 0.02537 |
| **T06E4.14** | hypothetical protein | [WBGene00077691](http://www.wormbase.org/db/gene/gene?name=WBGene00077691;class=Gene) | -0.76 | 0.02122 |
| **ugt-55** | UDP-GlucuronosylTransferase family member | [WBGene00011452](http://www.wormbase.org/db/gene/gene?name=WBGene00011452;class=Gene) | -0.76 | 0.04319 |
| **R12E2.7** | hypothetical protein | [WBGene00020033](http://www.wormbase.org/db/gene/gene?name=WBGene00020033;class=Gene) | -0.77 | 0.00281 |
| **col-144** | COLlagen family member (col-144) | [WBGene00000717](http://www.wormbase.org/db/gene/gene?name=WBGene00000717;class=Gene) | -0.77 | 0.00003 |
| **C16D9.1** | hypothetical protein | [WBGene00015857](http://www.wormbase.org/db/gene/gene?name=WBGene00015857;class=Gene) | -0.78 | 0.04052 |
| **col-172** | COLlagen family member (col-172) | [WBGene00000745](http://www.wormbase.org/db/gene/gene?name=WBGene00000745;class=Gene) | -0.79 | 0.02235 |
| **cyp-13A7** | CYtochrome P450 family member (cyp-13A7) | [WBGene00000372](http://www.wormbase.org/db/gene/gene?name=WBGene00000372;class=Gene) | -0.80 | 0.01954 |
| **grd-6** | GRounDhog (hedgehog-like family) family member | [WBGene00001695](http://www.wormbase.org/db/gene/gene?name=WBGene00001695;class=Gene) | -0.82 | 0.01125 |
| **Y106G6G.6** | hypothetical protein | [WBGene00013715](http://www.wormbase.org/db/gene/gene?name=WBGene00013715;class=Gene) | -0.84 | 0.00896 |
| **col-147** | COLlagen family member (col-147) | [WBGene00000720](http://www.wormbase.org/db/gene/gene?name=WBGene00000720;class=Gene) | -0.85 | 0.00006 |
| **dpy-5** | DumPY : shorter than wild-type family member | [WBGene00001067](http://www.wormbase.org/db/gene/gene?name=WBGene00001067;class=Gene) | -0.90 | 0.00000 |
| **E02A10.4** | hypothetical protein | [WBGene00008454](http://www.wormbase.org/db/gene/gene?name=WBGene00008454;class=Gene) | -0.90 | 0.01569 |
| **col-41** | COLlagen family member (col-41) | [WBGene00000618](http://www.wormbase.org/db/gene/gene?name=WBGene00000618;class=Gene) | -0.91 | 0.00000 |
| **glb-6** | GLoBin family member (glb-6) | [WBGene00015969](http://www.wormbase.org/db/gene/gene?name=WBGene00015969;class=Gene) | -0.91 | 0.03317 |
| **pqn-71** | Prion-like-(Q/N-rich)-domain-bearing protein | [WBGene00004153](http://www.wormbase.org/db/gene/gene?name=WBGene00004153;class=Gene) | -0.94 | 0.00001 |
| **sqt-1** | SQuaT family member (sqt-1) | [WBGene00005016](http://www.wormbase.org/db/gene/gene?name=WBGene00005016;class=Gene) | -0.94 | 0.00002 |
| **F46G11.1** | hypothetical protein | [WBGene00018514](http://www.wormbase.org/db/gene/gene?name=WBGene00018514;class=Gene) | -0.94 | 0.00732 |
| **F39D8.3** | hypothetical protein | [WBGene00009561](http://www.wormbase.org/db/gene/gene?name=WBGene00009561;class=Gene) | -0.94 | 0.03169 |
| **C05E7.2** | hypothetical protein | [WBGene00007344](http://www.wormbase.org/db/gene/gene?name=WBGene00007344;class=Gene) | -1.01 | 0.00080 |
| **col-17** | COLlagen family member (col-17) | [WBGene00000606](http://www.wormbase.org/db/gene/gene?name=WBGene00000606;class=Gene) | -1.04 | 0.00000 |
| **C45B2.2** | hypothetical protein | [WBGene00016659](http://www.wormbase.org/db/gene/gene?name=WBGene00016659;class=Gene) | -1.05 | 0.03681 |
| **C18H7.1** | hypothetical protein | [WBGene00015993](http://www.wormbase.org/db/gene/gene?name=WBGene00015993;class=Gene) | -1.06 | 0.00000 |
| **C10A4.5** | hypothetical protein | [WBGene00015666](http://www.wormbase.org/db/gene/gene?name=WBGene00015666;class=Gene) | -1.14 | 0.03198 |
| **col-169** | COLlagen family member (col-169) | [WBGene00000742](http://www.wormbase.org/db/gene/gene?name=WBGene00000742;class=Gene) | -1.14 | 0.00045 |
| **osm-9** | OSMotic avoidance abnormal family member | [WBGene00003889](http://www.wormbase.org/db/gene/gene?name=WBGene00003889;class=Gene) | -1.14 | 0.00289 |
| **Y45F10D.2** | hypothetical protein | [WBGene00012883](http://www.wormbase.org/db/gene/gene?name=WBGene00012883;class=Gene) | -1.15 | 0.00138 |
| **W08E12.6** | hypothetical protein | [WBGene00021087](http://www.wormbase.org/db/gene/gene?name=WBGene00021087;class=Gene) | -1.17 | 0.00098 |
| **col-168** | COLlagen family member (col-168) | [WBGene00000741](http://www.wormbase.org/db/gene/gene?name=WBGene00000741;class=Gene) | -1.18 | 0.02217 |
| **C01G10.6** | hypothetical protein | [WBGene00007233](http://www.wormbase.org/db/gene/gene?name=WBGene00007233;class=Gene) | -1.22 | 0.01714 |
| **Y47G6A.15** | hypothetical protein | [WBGene00021641](http://www.wormbase.org/db/gene/gene?name=WBGene00021641;class=Gene) | -1.36 | 0.02235 |
| **clec-165** | C-type LECtin family member (clec-165) | [WBGene00009523](http://www.wormbase.org/db/gene/gene?name=WBGene00009523;class=Gene) | -1.43 | 0.00251 |
| **F14B6.4** | hypothetical protein | [WBGene00008785](http://www.wormbase.org/db/gene/gene?name=WBGene00008785;class=Gene) | -1.53 | 0.03766 |
| **col-105** | COLlagen family member (col-105) | [WBGene00000679](http://www.wormbase.org/db/gene/gene?name=WBGene00000679;class=Gene) | -1.57 | 0.04721 |

**Suppl. Table 2: Functional classification of upregulated DEGs (FunCat)**

| **FunCat ID** | **Category** | **Hits** | **P-Value** |
| --- | --- | --- | --- |
| 16.03.03 | RNA binding | 14 | 0.000000 |
| 12.01.01 | ribosomal proteins | 11 | 0.000000 |
| 12.01 | ribosome biogenesis | 11 | 0.000005 |
| 01 | metabolism | 4 | 0.000015 |
| 77 | organ localization | 3 | 0.000603 |
| 77.03 | animal organ | 3 | 0.000603 |
| 10 | cell cycle and dna processing | 1 | 0.000727 |
| 18.02 | regulation of protein activity | 1 | 0.000728 |
| 36 | systemic interaction with the environment | 5 | 0.003068 |
| 18.02.01 | enzymatic activity regulation / enzyme regulator | 1 | 0.005647 |
| 32 | cell rescue, defense and virulence | 2 | 0.005713 |
| 30 | cellular communication/signal transduction mechanism | 9 | 0.006923 |
| 18 | regulation of metabolism and protein function | 3 | 0.007960 |
| 01.05 | C-compound and carbohydrate metabolism | 1 | 0.009235 |
| 12 | protein synthesis | 12 | 0.010946 |
| 16.19 | nucleotide/nucleoside/nucleobase binding | 3 | 0.019488 |
| 41 | development (systemic) | 10 | 0.019691 |
| 77.03.01 | nervous system | 2 | 0.022366 |
| 47 | organ differentiation | 1 | 0.022415 |
| 77.03.15 | reproductive apparatus | 1 | 0.022420 |
| 47.03 | animal organ | 1 | 0.022441 |
| 11.04.03.01.10 | regulation of splicing | 3 | 0.023530 |
| 36.25 | animal specific systemic sensing and response | 4 | 0.024538 |
| 30.05 | transmembrane signal transduction | 7 | 0.025171 |
| 41.05 | animal development | 8 | 0.026141 |
| 40.02 |  | 8 | 0.026337 |
| 40 | cell fate | 11 | 0.030278 |
| 77.03.01.01 | central nervous system | 2 | 0.033586 |
| 01.04 | phosphate metabolism | 1 | 0.034636 |
| 30.01.05 | enzyme mediated signal transduction | 1 | 0.035667 |
| 12.04 | translation | 9 | 0.036059 |
| 70.02 | eukaryotic plasma membrane / membrane attached | 5 | 0.043128 |
| 40.01 | cell growth / morphogenesis | 3 | 0.044270 |
|  |  |  |  |

**Suppl. Table 3:** Functional classification of upregulated DEGs (GO Term: Biological Process)

| **Term** | **Hits** | **P-Value** |
| --- | --- | --- |
| GO:0006412~translation | 12 | 0.000000 |
| GO:0007218~neuropeptide signaling pathway | 6 | 0.000003 |
| GO:0010467~gene expression | 16 | 0.000004 |
| GO:0034645~cellular macromolecule biosynthetic process | 14 | 0.000058 |
| GO:0009620~response to fungus | 3 | 0.000059 |
| GO:0031640~killing of cells of another organism | 3 | 0.000059 |
| GO:0050832~defense response to fungus | 3 | 0.000059 |
| GO:0009059~macromolecule biosynthetic process | 14 | 0.000060 |
| GO:0001906~cell killing | 3 | 0.000118 |
| GO:0051707~response to other organism | 3 | 0.000410 |
| GO:0044249~cellular biosynthetic process | 15 | 0.000466 |
| GO:0009058~biosynthetic process | 15 | 0.000789 |
| GO:0040007~growth | 15 | 0.001682 |
| GO:0009792~embryonic development ending in birth or egg hatching | 22 | 0.001857 |
| GO:0009790~embryonic development | 22 | 0.002373 |
| GO:0007275~multicellular organismal development | 26 | 0.002903 |
| GO:0040008~regulation of growth | 18 | 0.003013 |
| GO:0009607~response to biotic stimulus | 3 | 0.003572 |
| GO:0044267~cellular protein metabolic process | 13 | 0.004013 |
| GO:0048518~positive regulation of biological process | 18 | 0.004864 |
| GO:0032502~developmental process | 26 | 0.005160 |
| GO:0019538~protein metabolic process | 15 | 0.005238 |
| GO:0045927~positive regulation of growth | 17 | 0.005770 |
| GO:0051704~multi-organism process | 3 | 0.007948 |
| GO:0044260~cellular macromolecule metabolic process | 17 | 0.008106 |
| GO:0032501~multicellular organismal process | 27 | 0.008663 |
| GO:0043170~macromolecule metabolic process | 19 | 0.009253 |
| GO:0007186~G-protein coupled receptor protein signaling pathway | 6 | 0.013069 |
| GO:0040010~positive regulation of growth rate | 15 | 0.014592 |
| GO:0040009~regulation of growth rate | 15 | 0.014665 |
| GO:0002119~nematode larval development | 15 | 0.017480 |
| GO:0002164~larval development | 15 | 0.017564 |
| GO:0048519~negative regulation of biological process | 6 | 0.018910 |
| GO:0009791~post-embryonic development | 15 | 0.019129 |
| GO:0000003~reproduction | 16 | 0.020591 |
| GO:0051239~regulation of multicellular organismal process | 8 | 0.021683 |
| GO:0042742~defense response to bacterium | 2 | 0.026741 |
| GO:0009617~response to bacterium | 2 | 0.026741 |
| GO:0007166~cell surface receptor linked signal transduction | 6 | 0.030428 |
| GO:0040014~regulation of multicellular organism growth | 6 | 0.037239 |
| GO:0006952~defense response | 3 | 0.038405 |
|  |  |  |
